# Fibroblastic reticular cells of the lymphoid tissues modulate T cell activation threshold during homeostasis via hyperactive cyclooxygenase-2/prostaglandin E_2_ axis

**DOI:** 10.1038/s41598-017-03459-5

**Published:** 2017-06-13

**Authors:** Miao Yu, Gang Guo, Xin Zhang, Li Li, Wei Yang, Roni Bollag, Yan Cui

**Affiliations:** 1 Department of Biochemistry and Molecular Biology, Cancer Immunology, Inflammation & Tolerance Program, Georgia Cancer Center, Augusta University, Augusta, GA 30912 USA; 2 Institution of Translational Research, Gayle & Tom Benson Cancer Center, 1N505A, Ochsner Clinic Foundation, 1514 Jefferson Highway, New Orleans, LA 70121 USA; 3 Tumor Tissue and Serum Biorepository, Georgia Cancer Center, Augusta University, Augusta, GA 30912 USA; 4 Department of Immunology, College of Basic Medical Sciences, Norman Bethune Health Science Center, Jilin University, 126 Xinmin Avenue, Changchun, 130021 China

## Abstract

Fibroblastic reticular cells (FRCs) in the T cell zone of lymph nodes (LNs) are pivotal for T cell survival, mobility, and peripheral tolerance. Here, we demonstrate that during homeostasis, FRCs also suppress T cell activation via producing high level of prostaglandin E_2_ (PGE_2_) due to their thousands-fold higher cyclooxygenase-2 (COX-2) expression than immune cells. This hyperactive COX-2/PGE_2_-induced suppression is evident during antigen-specific and non-antigen-specific activations. It is implicated as suppressed TCR-signaling cascades, reduced alterations in activation markers, and inhibited cytokine production of freshly isolated T cells or T cells co-cultured with FRCs compared with those cultured without FRCs. Different from T cell dysfunction, this FRC-mediated suppression is surmountable by enhancing the strength of stimulation and is reversible by COX-2 inhibitors. Furthermore, T cells in the FRC environment where *Cox-2* is genetic inactivated are more sensitive and rapidly activated upon stimulations than those in WT environment. Significantly, FRCs of human lymphoid organs manifest similar COX-2/PGE_2_ hyperactivity and T cell suppression. Together, this study identifies a previously unappreciated intrinsic mechanism of FRCs shared between mice and humans for suppressing T cell sensitivity to activation via PGE2, underscoring the importance of FRCs in shaping the suppressive milieu of lymphoid organs during homeostasis.

## Introduction

Secondary lymphoid organs, such as lymph nodes (LNs), are pivotal for host immunity where a complex network of non-hematopoietic stromal cells organizes immune cells into distinct compartments^1–3^. The stromal network provides structural and functional environment to modulate immune cell survival and mobility^2–5^. Fibroblastic reticular cells (FRCs) are an important subset of stromal cells that serve as the backbone of the interconnected reticular conduit network in the T cell zone^2, 5–7^. It has been increasingly appreciated that the FRC network plays crucial roles in supporting T cell survival, modulating T cell and dendritic cell mobility, and regulating the balance between T cell activation and tolerance via producing cytokines/chemokines and transporting growth factors and soluble antigens^2, 4, 6–8^. Recently, compelling evidence demonstrated that FRCs are capable of presenting peripheral tissue–restricted antigens (PTAs) to enforce peripheral T cell tolerance by deleting self-reactive T cells^9–12^. Moreover, during inflammation or following immune activation, FRCs also actively suppress T cell proliferation by producing nitric oxide (NO), which is resulted by the effector cytokine-stimulated upregulation of inducible nitric oxide synthase (iNOS)^13–15^. This iNOS/NO-mediated T cell suppression facilitates the re-establishment of homeostasis during inflammation^13, 15, 16^. While these observations clearly underscore the crucial function of FRCs in regulating immune response via multiple mechanisms, it remains unclear whether additional undiscovered mechanisms also contribute to FRC-mediated immune regulation or restraint of T-cell activation.

The capacity of host immune system in maintaining self-tolerance, while remaining rapidly responsive against external threats to control pathogenic invaders, has been a fundamental issue of extensive investigation^17–20^. It is now known that enforcement of tolerance is achieved via multiple mechanisms and is regulated at various levels, including deletion of auto-reactive T cells, stringent immune suppression during homeostasis or under pathological conditions, and restraint of excessive activation of self-damaging T cells by temporally and moderately fine-tuning T cell activation signal or modulating activation threshold during homeostasis^17, 19–25^. Seminal studies illustrated that regulatory T cells (Treg)^26–28^, regulatory dendritic cells (DCs)^21, 29^, and myeloid derived suppressor cells^24, 25^ impose immunosuppression to rigorously inhibit T cell activation and proliferation either via cell-cell contact or through soluble factors^17, 19–22^.

Prostaglandin E_2_ (PGE_2_), which is a metabolite of arachidonic acid generated sequentially by cyclooxygenase-1 (COX-1) or COX-2 (also known as prostaglandin-endoperoxide synthase 2, PTGS2) and PGE synthase (PGES)^30, 31^, is a small molecule known to suppress T cell activation^23, 30, 32, 33^. Tumor immunology studies showed that the COX-2/PGE_2_ pathway is exploited by tumors and myeloid derived suppressor cells (MDSCs) within the tumor microenvironment (TME) as a mechanism of immune evasion and a high expression level of *Cox-2*, associated with high level of PGE_2_ production, in these cells is correlated positively with their capacity of T cell suppression^23, 33, 34^. However, it is yet unknown whether PGE_2_ directly participates in the maintenance of T cell tolerance during homeostasis.

Here, we demonstrated that COX-2/PGE_2_ activity in FRCs serves as another mechanism of FRC-mediated peripheral T cell tolerance. Particularly, the COX-2/PGE_2_ pathway is hyperactived in FRCs during homeostasis that is at least thousands-fold higher than that in lymphoid and myeloid subpopulations, which reduces the magnitude of TCR-signaling and elevates T cell activation threshold by both antigen-specific and non-antigen-specific TCR-ligation. Unlike T cell dysfunction, this FRC-mediated T cell suppression can be overcome by enhanced strength of activation stimuli and is reversible by COX-2 inhibitors or the genetic inactivation. Importantly, FRCs of human lymphoid tissues share similar hyperactivated COX-2/PEG2 pathway, suggesting a general intrinsic mechanism of FRCs in modulating T cell activation threshold during homeostasis via producing PGE_2_.

## Results

### FRCs from peripheral lymph nodes of naïve mice suppress T cell activation via an iNOS/NO-independent mechanism

**Figure 1:**
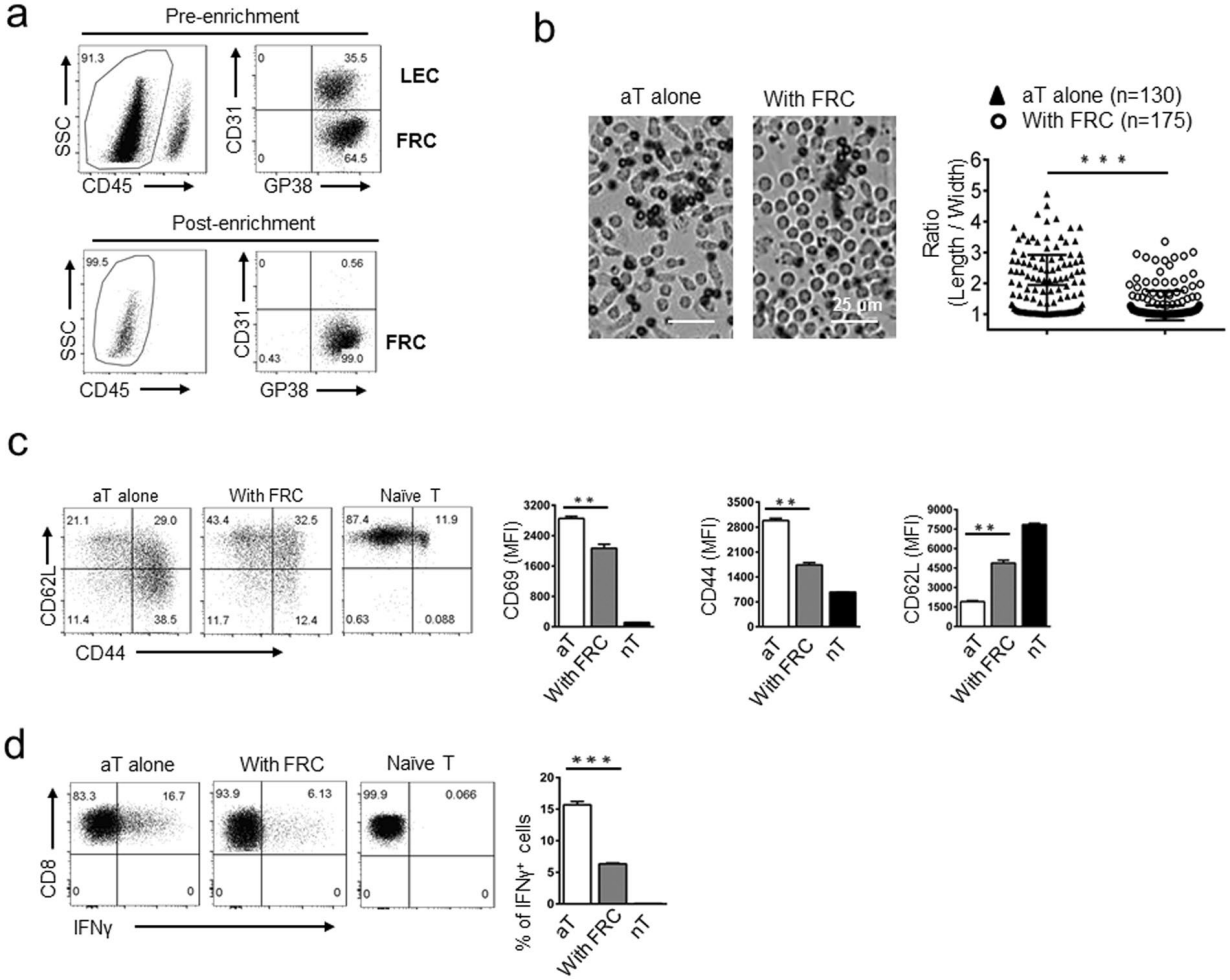
FRC-mediated suppression of T cell activation during early activation phase is iNOS/NO independent. (**a**) Representative flow cytometry profiling of *ex vivo* expended SLN stromal populations and FRC purification via FACsorting as CD45^−^GP38^+^CD31^−^ cells. (**b**) Morphological changes of activated T cells 20 hrs post-activation by anti-CD3/CD28 beads (black) in the absence (left) or presence (right) of FRCs were examined microscopically. The elongation of individual activated T cell was calculated as the ratio of length/width measured by a computer program from three independent pictures. Scale bars, 25 μm. (**c**) Naïve T cells were stimulated by anti-CD3/CD28 beads for 15 hrs, in the absence or presence of SLN-FRCs. T cell activation was assessed by flow cytometry as changes in surface expression of CD69, CD62L, and CD44. Numbers in quadrants indicate cell percentages. MFI values of CD69, CD44 and CD62L are summarized in bar chart. (**d**) Following T cell activation by to anti-CD3/CD28 beads in the absence or presence of FRCs for 16 hrs, brefeldin A (BFA, 5 μg/ml) was added to T cells for 4 hrs. CD8 T cell production of IFN-γ was determined via intracellular staining followed by FACS analysis. Data from three independent experiments are summarized in bar chart. **p < 0.01, ***p < 0.001 (nonparametric Mann-Whitney test). Data are representative of two to three independent experiments with three replicates each. Error bars depict mean ± SEM.

Recent studies demonstrated that FRCs of the skin draining lymph nodes (SLNs) play an important role in maintaining peripheral T cell tolerance via presenting PTAs and elevating NO production^11, 12, 14, 15, 35, 36^. To explore whether FRCs employ additional, as yet unidentified, mechanisms to maintain T cell tolerance during homeostasis, we purified primary SLN-FRCs via FACSorting as CD45^−^gp38^+^CD31^−^ cells as described in the Methods (Fig. 1a). Similar to previous reported^13^, these FRCs suppressed T cell proliferation stimulated by anti-CD3/CD28 beads at a ratio of 1 FRC to 25 T cells (Fig. S1a). Analysis of NO production by FRCs confirmed their minimal production during homeostasis, which was below the limit of detection (Fig. S1b). Upon T cell activation, NO level in FRC-T cell co-culture reached 1 μM by 24 hours and 25 μM by 48 hours (Fig. S1b), similar to the previous report^13^. These results suggest that the iNOS/NO-pathway was inactive in FRCs during homeostasis or within 24 hours of T cell activation. Thus, we focused our investigation on changes occur within 24-hours of T cell activation to clarify the existence of an iNOS/NO-independent suppressive mechanism. Microscopically, by 20 hours of anti-CD3/CD28 activator stimulation, T cells were overtly elongated in the absence of FRCs (Fig. 1b), which was markedly suppressed by the presence of FRCs (Fig. 1b, quantified as the ratio of T cell length/width). Flow cytometry analysis of cellular forward scatter (FSC) also confirmed FRC-mediated T cell suppression, which could not be reversed by a NOS inhibitor NG-monomethyl L-arginine (L-NMMA) (Fig. S1c). Examination of changes in cell surface markers, including up-regulation of CD69 and CD44 and down-regulation of CD62L, revealed that the presence of FRCs greatly inhibited CD69 and CD44 up-regulation, as well as CD62L shedding in CD8 (Fig. 1c) and CD4 T cells (Fig. S1d) at 15 hours post-activation. Functional analysis of intracellular cytokine staining demonstrated that FRCs markedly inhibited T cell effector cytokine IFN-γ (Fig. 1d) and IL-2 (Fig. S1e) production in activated CD8 and CD4 T cells, respectively. Collectively, these results demonstrate that steady-state FRCs, i.e. unperturbed by inflammatory cytokines, suppress T cell activation in a non-antigen-specific manner, independent of the iNOS-NO pathway. We propose that this observed FRC-mediated suppression of T cell activation likely reflects an intrinsic property of FRCs *in vivo* during homeostasis for restraining T cells from accidental activation.

### FRC-mediated inhibition of T cell activation occurs both *in vivo* and *in vitro* and is reversible

**Figure 2:**
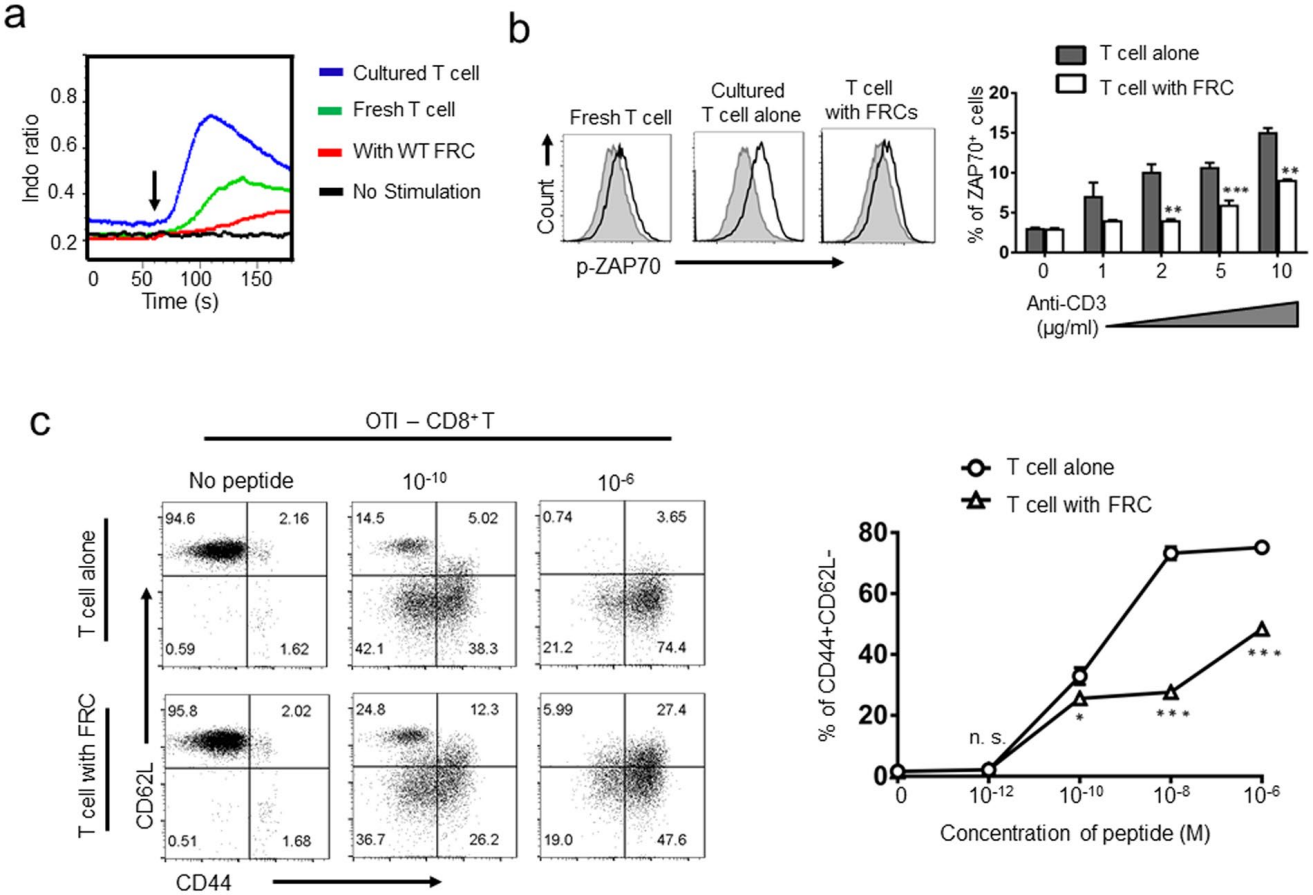
FRCs suppress antigen-specific and non-antigen-specific T cell activation and subsequent effector cytokine production. (**a**) Naïve T cells, either immediately following isolation from LNs (green line), cultured overnight in the absence of FRCs (blue), or in the presence of FRCs (red), respectively, were first loaded with 5 µM of Indo 1-AM dye, labeled with biotinylated anti-CD3 (1 μM) for 15 min, and subsequently stimulated by addition of Streptavidin (10 mg/ml) to trigger TCR-ligation. Calcium-flux was examined via Flow cytometry. Arrow indicates the addition of streptavidin. Data are representative of three independent experiments. (**b**) Following 30 min of anti-CD3 (1–10 μg/ml) mediated stimulation, ZAP70 phosphorylation in T cells was determined via flow cytometry analysis following intracellular staining. The representative histograms of ZAP70 phosphorylation in 2 μg/ml of anti-CD3 stimulated freshly isolated T cells (left), T cells cultured overnight alone (middle), or co-cultured with FRC (left), respectively (top row, dark lines), were compared with unstimulated controls (gray lines). (**c**) OT-I splenic cells were stimulated by varying concentrations of SIINFEKL peptide in the absence or presence of SLN-FRCs. OT-I splenic cells cultured in the absence of SIINFEKL peptide were used as non-activation control. T cell activation at 20 hrs post-stimulation was assessed as changes in CD62L and CD44 expression via flow cytometry. Data are representative of three independent experiments with three replicates each. Error bars depict mean ± SEM. *p < 0.05, **p < 0.01, ***p < 0.001 compared with respective control (nonparametric Mann-Whitney test).

Because T cell-FRC contact occurs constantly *in vivo*, we reasoned that freshly isolated T cells should retain their FRC-imposed suppression at least within a short period of time following isolation if this FRC-mediated suppression exists *in vivo*. Conversely, this suppression will be alleviated in T cells cultured *ex vivo* in the absence of FRCs, which should become more responsive to activation stimuli. Because immediately following TCR ligation, the strength of T cell activation signal can be monitored by and is directly correlative to the level of calcium (Ca^2+^)-flux and Zap70 phosphorylation^37, 38^, we employed specific assays to examine the level of T cell activation following anti-CD3 mediated TCR-ligation. As expected, T cells cultured in the absence of FRCs for 12 hours, but supplemented with recombinant murine IL-7 to maintain viability, responded rapidly to anti-CD3 stimulation with a higher level of Ca^2+^-flux (Fig. 2a, blue line) than that of freshly isolated T cells, which remained in constantly contact with FRCs *in vivo* (Fig. 2a, green line). More strikingly, T cells cultured *ex vivo* with FRCs for 12 hours manifested a further reduction in Ca^2+^-flux following anti-CD3-TCR-ligation, supporting the notion of FRC-mediated suppression of TCR-signaling (Fig. 2a, red line). Likewise, examination of Zap70 phosphorylation also confirmed that removal of FRCs from T cell culture led to elevated TCR-signaling compared with those of freshly isolated naïve T cells and T cells cultured in the presence of FRCs (Fig. 2b). To determine whether the FRC-mediated suppression of T cell signaling is overcomable by enhancing the strength of TCR-ligation, we stimulated T cells with varying concentrations of anti-CD3. Associated with an increasing level of anti-CD3, Zap70 phosphorylation was increased gradually (Fig. 2b). When anti-CD3 was elevated to the range of 5–10 μg/ml, Zap70 phosphorylation in T cells co-cultured with FRCs reached a level comparable to that in T cells cultured in the absence of FRCs that was stimulated by 1 μg/ml anti-CD3 (Fig. 2b). Further analysis of anti-CD3 binding to cultured CD4 and CD8 T cells at various antibody concentrations showed similar level of CD3-FITC binding regardless of the absence or presence of FRCs (Fig. S2). These results suggest that our observed FRC-mediated suppression of TCR-signaling is not resulted by the interference of FRCs to TCR-binding.

To investigate whether FRC-mediated inhibition of T cell activation also occurs during antigen-specific activation, we stimulated OVA-antigen-specific OT-I CD8 T cell with antigen presenting cells (APCs) loaded with a wide range of OT-I peptide, which was previously defined to show dose-dependent T cell activation^39^. Compared with the OT-I cells cultured in the absence of FRCs, the activation of OT-I T cells cultured in the presence of FRCs was suppressed in most of the doses tested (Fig. 2c). Nevertheless, those OT-I cells could still be activated, which required about 100-fold higher antigen concentration to achieve a comparable activation of the OT-I cells cultured in the absence of FRCs (Fig. 2c). Together, these results suggest that during homeostasis, T cell-FRC interaction suppresses T cell responses to activation stimuli such that a more stringent activation signal is required to achieve a similar level of T cell activation. This likely represents another yet to be defined mechanism of FRC-mediated T cell tolerance that restrains T cells from accidental activation during minor environmental perturbations.

### FRCs suppress T cell activation through heat-stable soluble factors, which can be reversed by inhibitors of cyclooxygenases

**Figure 3:**
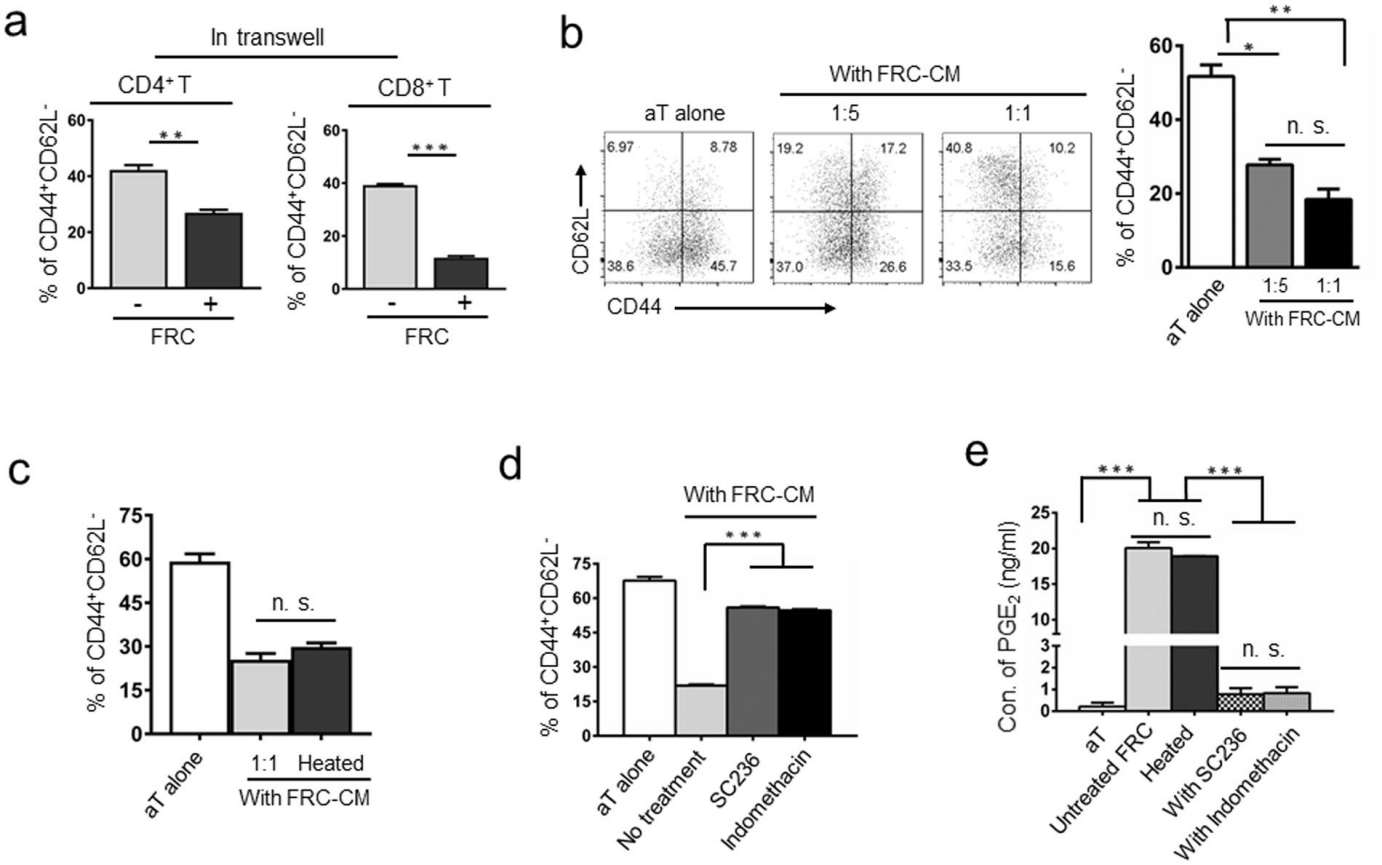
FRCs suppress T cell activation through secretion of heat-stable soluble factors. (**a**) T cells were activated in transwell plates where they were placed with activation beads in the upper chamber and separated from FRCs in the lower compartment by a permeable membrane (1.0 μm). T cell activation was assessed by flow cytometry as changes in the expression of CD44 and CD62L. (**b**) FRC-conditioned medium (FRC-CM), supernatant harvested from a FRC overnight-culture, was added to T cell activation assay at the dilution of 1:1 or 1:5. The percentage of activated CD44^+^CD62L^−^ cells is summarized as bar chart. (**c**) FRC-CM was heated to 100 °C for 5 min. The chilled medium was added to T cell activation assay at the dilution of 1:1. n.s. = not significant. (**d**) FRC-CM from the FRC culture in the presence of COX-2 selective inhibitor SC236 (5 μM) or COX-2 non-selective inhibitor indomethacin (1 μM) for 24 h were collected and added to T cell activation assay at 1:1 dilution. The percentage of activated CD44^+^CD62L^−^ cells was determined via flow cytometry. (**e**) PGE_2_ concentration in the supernatant of activated T cells and FRC-CM in the absence or presence of various inhibitors was determined via ELISA. Data (**a**–**e**) are representative of two to three independent experiments (mean ± SEM; *P < 0.05, **P < 0.01, ***P < 0.001; nonparametric Mann-Whitney test).

Multiple molecular mechanisms have been implicated in suppressing T cell activation and maintaining peripheral tolerance^13, 23–25, 40^. To examine whether this FRC-mediated T cell suppression requires cell-cell contact, we employed a trans-well culture system where T cells were activated in the upper chamber and separated from FRCs in the lower compartment that only allowed the exchange of soluble factors. As shown in Fig. 3a, in the absence of direct cell-cell contact, FRCs retained their capacity of suppressing T cell activation, which was demonstrated by inhibition of CD44^+^CD62L^−^ subpopulation. This result suggests that during homeostasis, FRCs suppress T cell activation via soluble factors, which is different from the reported iNOS/NO-mediated, cell contact-dependent T cell suppression during inflammation^13, 16^. Addition of filtered FRC-conditioned medium (FRC-CM) to T cell activation culture induced a dose-dependent suppression of T cell activation (Fig. 3b), further confirming the contribution of a soluble factor(s). To assess whether this inhibitory soluble factor(s) is a heat-stable small molecule or heat-sensitive molecule, such as protein or peptide, we treated the FRC-CM to 100 °C for 5 minutes before adding it to T cell activation assay. Interestingly, heat treatment did not alleviate the inhibitory effect of FRC-CM on T cell activation (Fig. 3c), suggesting the likelihood of this heat-resistant inhibitory factor(s) being small molecule(s).

Small molecules, including NO, adenosine (ADO), and PGE_2,_ as well as Arginase (Arg) and indoleamine 2,3-dioxygenase (IDO) metabolites, are known inhibitors of T cell activation^13, 23–25, 40, 41^. Early studies excluded the potential involvements of Arg and IDO in FRC-mediated immune suppression^13, 16^ and our aforementioned results ruled out the involvement of NO during homeostasis. We then examined the effects of blocking the ADO pathway either via A2AR and A2BR antagonists (ZM241385 and PSB1115, respectively) or an inhibitor of CD73 (APCP) to prevent ADO generation^41–43^. Our results demonstrated that none of these inhibitors reversed the FRC-mediated inhibition of T cell activation, implying that this FRC-mediated suppression during homeostasis is independent of the ADO pathway (Fig. S3a). Further investigation of the potential involvement of PGE_2_ via treating FRCs with inhibitors of PGE_2_ synthesis, either a COX-2 specific inhibitor (SC-236) or a broad spectrum inhibitor of COX-1 and COX-2 (Indomethacin)^44^, demonstrated that FRC-mediated T cell inhibition was alleviated by either inhibitor (Fig. 3d). Measurement of PGE_2_ concentration in the FRC-CM confirmed a high level of PGE_2_ production by FRCs, up to 20 ng/ml/2 × 10^4^ FRCs/24 hrs (Fig. 3e), which was not affected by the heat-treatment of FRC-CM, but markedly reduced by addition of COX inhibitors to FRC culture (Fig. 3e). These results provide direct evidence of the hyperactive COX2-PGE_2_ axis as a mechanism of FRC-mediated T cell suppression during homeostasis. Furthermore, inclusion of PGE_2_ at varying concentrations (0–100 ng/ml) in T cell activation culture caused a dose-dependent inhibition of T cell activation, reminiscent to FRC-mediated inhibition (Fig. S3b). Likewise, presence of 20 ng/ml of PGE_2_ during early activation induced a similar level of suppression of TCR-ligation-induced Zap70 phosphorylation to that imposed by FRCs (Fig. S3c). Collectively, our results strongly support the notion that during homeostasis, FRCs suppress T cell activation through COX-2-mediated production of PGE_2_, which elevates the required minimal level of stimulation for T cell activation.

### Hyperactivated COX-2/PGE_2_ axis is an intrinsic property of SLN-FRCs during homeostasis *in vivo* and under standard culture condition

**Figure 4:**
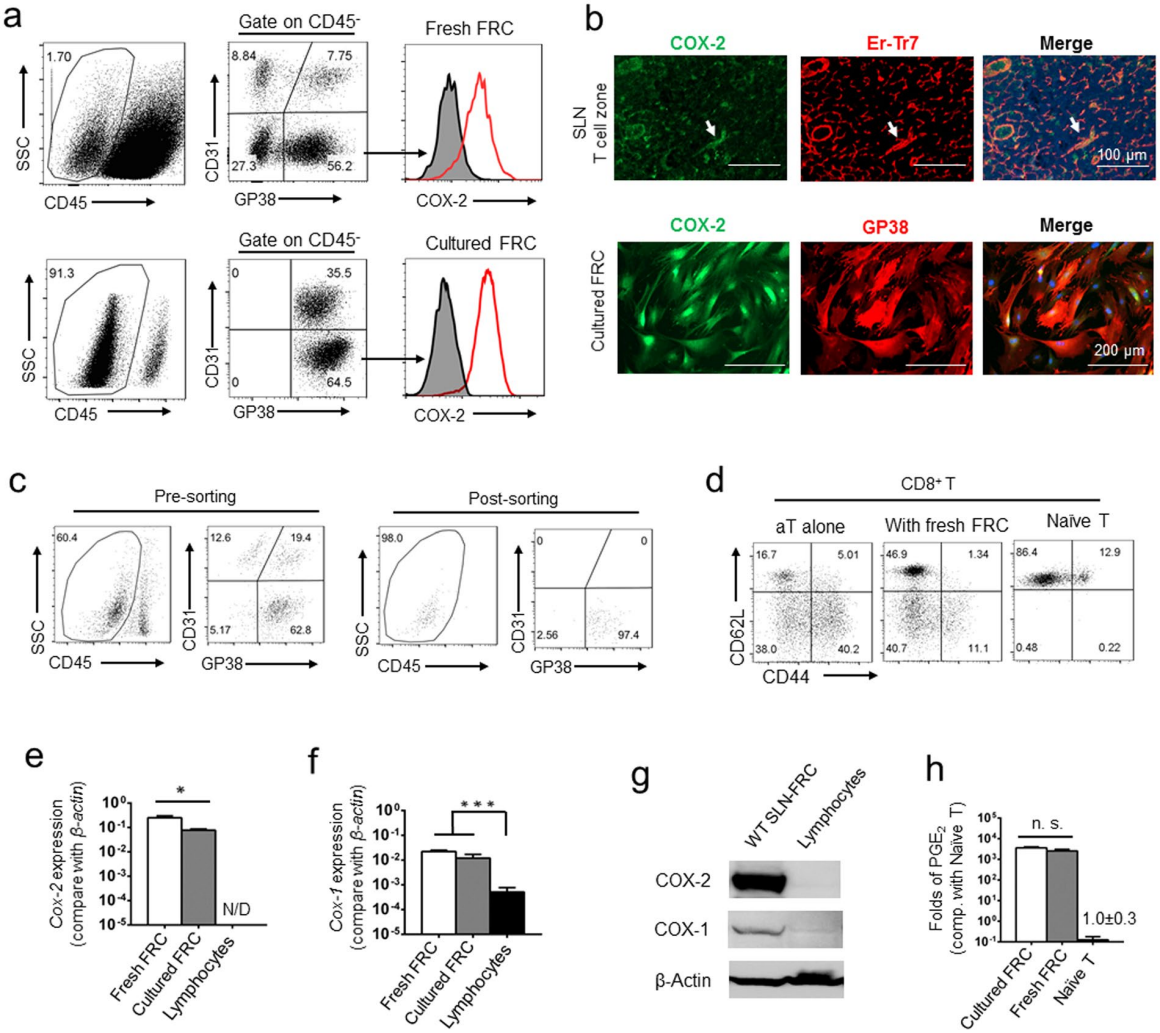
Hyperactive COX-2/PGE_2_ axis is an intrinsic property of FRC *in situ* during homeostasis. (**a**) Flow cytometry analysis of COX-2 expression in freshly isolated and cultured SLN-FRCs following intracellular staining (red line) compared with isotype control (gray). (**b**) Immunofluorescence staining of COX-2 (green) and Er-Tr7 (red) in cryosection of SLN (top row) and cultured FRCs (bottom row) with COX-2 (green) and GP38 (red), overlaying with nuclear DAPI staining (blue). Scale bars, 100 μm (top row) and 200 μm (bottom rows), respectively. (**c**) Sorting strategy of freshly isolated SLN-FRCs (CD45^−^CD31^−^GP38^+^). (**d**) Isolated CD8^+^ T cells were stimulated by anti-CD3/28 activation beads with or without the condition medium from freshly isolated SLN-FRC. T cell activation was assessed by flow cytometry analysis of CD62L and CD44. (**e**,**f**) Comparative analysis of *Cox-2* and *Cox-1* mRNA expression in freshly isolated FRCs, lymphocytes and cultured FRCs via quantitative real-time RT-PCR, which is normalized against the *b-actin* mRNA level of each sample. N/D, not detectable. (**g**) Cell lysates of cultured FRCs and fresh lymphocytes were subjected to SDS-PAGE gel and Western Blotting analysis to determine COX-2 and COX-1 protein expression using b-actin as the sample loading control. Cropped images of representative experimental results and the uncropped images are presented in Supplementary Fig. 7. (**h**) The accumulation of PGE_2_ in the supernatant of freshly isolated and cultured FRCs was compared with that of naïve T cells. The level of PGE_2_ is normalized as the production by 1 × 10^6^ cells/1 ml/24 h. *P < 0.05, ***P < 0.001, n.s. = not significant. Nonparametric Mann-Whitney test. Data are representative of two to three independent experiments (mean ± SEM).

To verify that the COX-2/PGE_2_ pathway is similarly activated in the SLN-FRCs *in vivo* during homeostasis, we further examined COX-2 expression in freshly isolated FRCs as compared with that of cultured FRCs. FACS analysis following intracellular staining and immunofluorescence staining (IF) *in situ* confirmed similar levels of COX-2 protein in fresh SLN-FRCs to those cultured for 7 days (Fig. 4a and b). To assess the suppressive function of FRCs during homeostasis and quantify their COX-2 expression, we FACSorted fresh SLN-FRCs from WT mice following the established protocol (Fig. 4c)^45, 46^. As expected, fresh FRCs suppressed T cell activation-associated changes in the expression of CD44 and CD62L in CD8 T cells as effectively as, if not more than, *ex vivo* expanded FRCs (Figs 4d and 1c). Strikingly, quantitative real-time RT-PCR analysis revealed that the *Cox-2* mRNA levels in both fresh and cultured FRCs were extremely high, about one-half or one-fiftieth of the mRNA level of the house keeping gene *β-actin*, respectively (Fig. 4e). In contrast, *Cox-2* mRNA was not detectable in lymphocytes harvested from SLNs (Fig. 4e). Early studies demonstrated that tumor associated myeloid-derived suppressor cells (MDSCs) express a high level of COX-2 as one of immunosuppressive mechanisms^23, 33, 47^. Comparative real-time RT-PCR analysis showed that the *Cox-2* mRNA levels in bone marrow-derived (BM)-MDSCs and BM-dendritic cells (BMDCs) was hundreds or thousands folds lower than that in FRCs (Fig. S4a).

In addition to COX-2, PGE_2_ production is also cooperatively regulated by the expression of mPges-1^30–32^. Furthermore, PGE_2_ secretion to the extracellular space depends on the prostaglandin transporter (PGT)^30–32^. We then analyzed and confirmed the extremely high expression levels of *mPges-1* and *Pgt* in FRCs via quantitative real-time RT-PCR as compared with those in lymphocytes (Fig. S4b and S4c). In contrast to the *Cox-2* expression, *Cox-1* mRNA level was not restricted to FRCs and was detectable in BM-DCs, BM-MDSCs, and lymphocytes (Figs 4f and S4a). The differential expression of COX-2 and COX-1 proteins in SLN-FRCs and lymphocytes was verified via Western Blotting (WB) analysis (Figs 4g and S4d), which also agreed with the tens of thousands-fold difference in their capacity of PGE_2_ production (Fig. 4h). Collectively, these results suggest that FRCs produce and maintain a high level of PGE_2_ in the extracellular space in a highly tissue-specific manner via elevated generation and secretion. Moreover, this extremely high level of PGE_2_ production by FRCs is dominantly contributed by the highly active COX-2 because COX-1 was similarly expressed among FRCs and myeloid cells, including BMDCs and MDSCs. This hyperactivated COX-2/PGE_2_ pathway is an intrinsic and previously unappreciated unique property of SLN-FRCs responsible for programing the suppressive milieu of lymphoid organs during homeostasis.

### Inactivation of the COX-2/PGE_2_ pathway in the FRCs of *Cox-2* mutant mice is associated with elevated T cell sensitivity to activation stimuli

**Figure 5:**
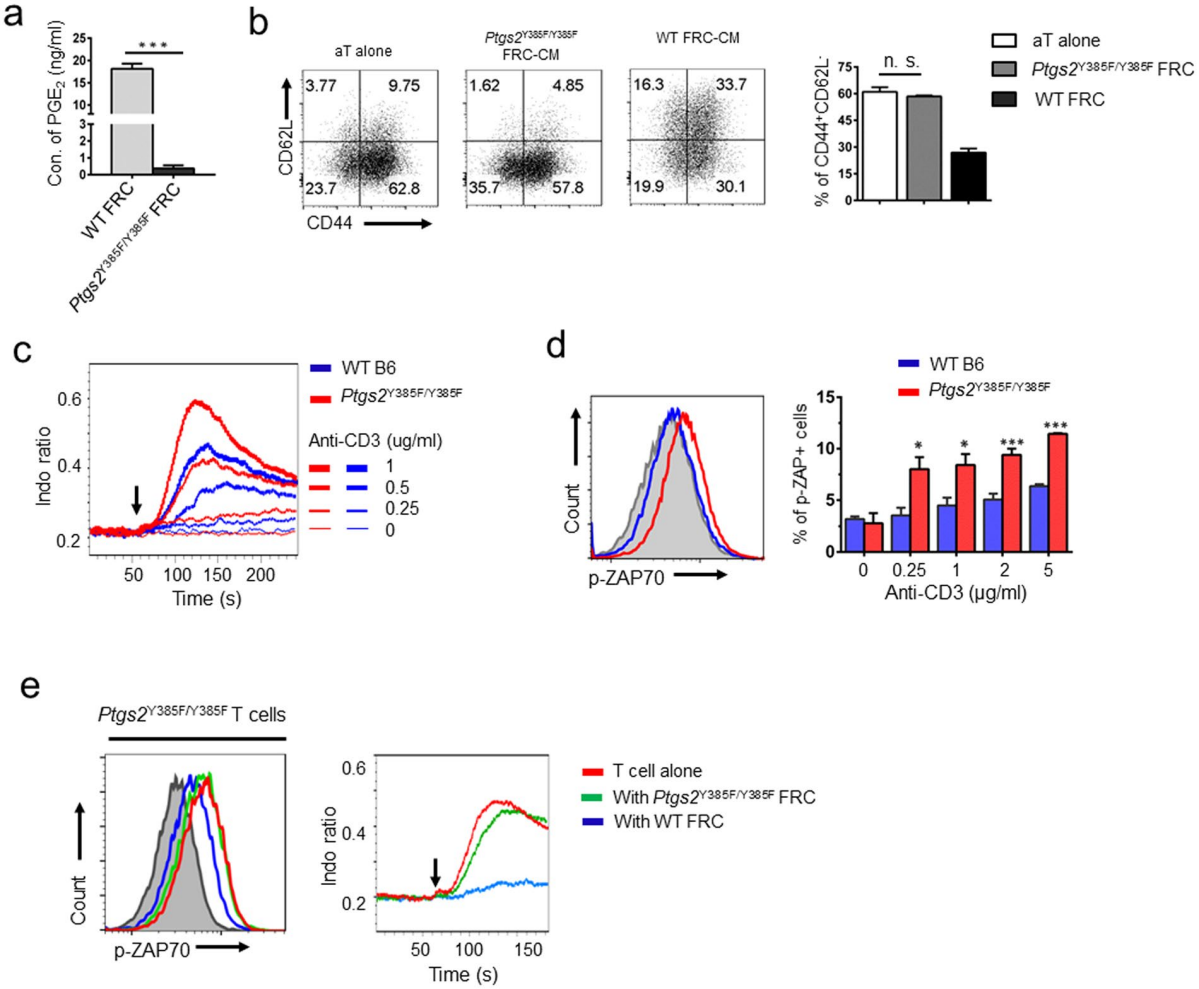
Hyperactive COX-2/PGE_2_ pathway in SLN-FRCs is associated with suppressed TCR-signaling cascade upon activation, which is reversible by genetic inactivation of *Cox-2*. (**a**) PGE_2_ concentration in the supernatant of WT and *Ptgs2* ^Y385F/Y385F^ FRCs following 24 hrs culture was examined. (**b**) FRC-CM of *C57BL/6* and *Ptgs2* ^Y385F/Y385F^ mice was added to T cell activation culture at a dilution of 1:1. The effects on T cell activation were examined via changes in CD44 and CD62L expression. (**c**) Freshly isolated T cells from C57BL/6 WT or *Ptgs2* ^Y385F/Y385F^ mice were used to determine their response to various level of anti-CD3 (0.25–1 μg/ml) stimulation as calcium-flux via flow cytometry. (**d**) ZAP70 phosphorylation in freshly isolated T cells of C57BL/6 WT and *Ptgs2* ^Y385F/Y385F^ mice following anti-CD3 (0.25–5 μg/ml) stimulation was examined via flow cytometry. Representative histogram of ZAP70 phosphorylation upon 2 μg/ml anti-CD3 stimulation is shown, where unstimulated T cells (gray) was used as a negative control. (**e**) CD4^+^ T cells from *Ptgs2* ^Y385F/Y385F^ mice were cultured overnight in the absence or presence of FRCs from C57BL/6 or *Ptgs2* ^Y385F/Y385F^ mice. T cells were then stimulated with anti-CD3 (1 μg/ml) and subjected to ZAP70 phosphorylation analysis (left) or calcium-flux assay (right). Arrow indicates the starting point of stimulation. Data (**a**–**e**) are representative of two to three independent experiments for CD4^+^ T cells (mean ± SEM; *P < 0.05, ***P < 0.001, n.s. = not significant; Nonparametric Mann-Whitney test).

If the COX2/PGE_2_ axis in SLN-FRCs is crucial in maintaining T cell tolerance or restricting T cell responses to environmental cues, COX-2 inactivation should lead to T cell hypersensitivity. To test this, we harvested SLN-FRCs and T cells from *Cox-2* mutant (*Ptgs2* ^*Y385F/Y385F*^, *Ptgs2* ^*M*^) mice, in which *Cox-2* incurs a point mutation that does not affect the expression of COX-2 protein, but inactivates its enzymatic activity for PGE_2_ production^48^. Despite similar level of *Cox-2* and *Cox-1* mRNA expression in *Ptgs2* ^*M*^ and WT FRCs (Fig. S5a), *Ptgs2* ^*M*^ FRCs produced minimal PGE2 (Fig. 5a). Therefore, unlike FRC-CM of WT mice, *Ptgs2* ^*M*^ FRC-CM was incapable of suppressing T cell activation (Fig. 5b), which agreed the observed loss of suppression of T cell activation in *Ptgs2* ^*M*^-FRC-T cell co-culture (Fig. S5b). Comparative analysis of fresh T cells from *Ptgs2* ^*M*^ mice to TCR-ligation induced Ca^2+^-flux and Zap70 phosphorylation demonstrated their increased sensitive to T cell activation stimulation as compared with those of fresh T cells from WT mice (Fig. 5c and d). Interestingly, the observed *Ptgs2* ^*M*^ T cell hypersensitivity compared with WT T cells could be suppressed following co-culture with WT-FRCs, but not *Ptgs2* ^*M*^-FRCs (Fig. 5e), to the same degree as those of WT T cells co-cultured with WT FRCs (Fig. S5c). Together, these results support our notion that basal PGE_2_ production by WT-FRCs was dominantly contributed by COX-2, but not COX-1. They further demonstrate that the observed *Ptgs2* ^*M*^ T cell hypersensitivity was resulted by the loss of FRC-PGE_2_ production, but not an intrinsic defect of *Ptgs2* ^*M*^ T cells. Therefore, during homeostasis, T cell responsiveness to TCR-mediated activation is likely dictated by the activity of the FRC-COX-2/PGE_2_ pathway that regulates the milieu of the lymphoid tissues and T cell activation threshold.

### Inactivation of the COX-2/PGE_2_ pathway in the FRCs of *Cox-2* mutant mice alters the kinetics of T cell response to antigen-specific DC vaccine

**Figure 6:**
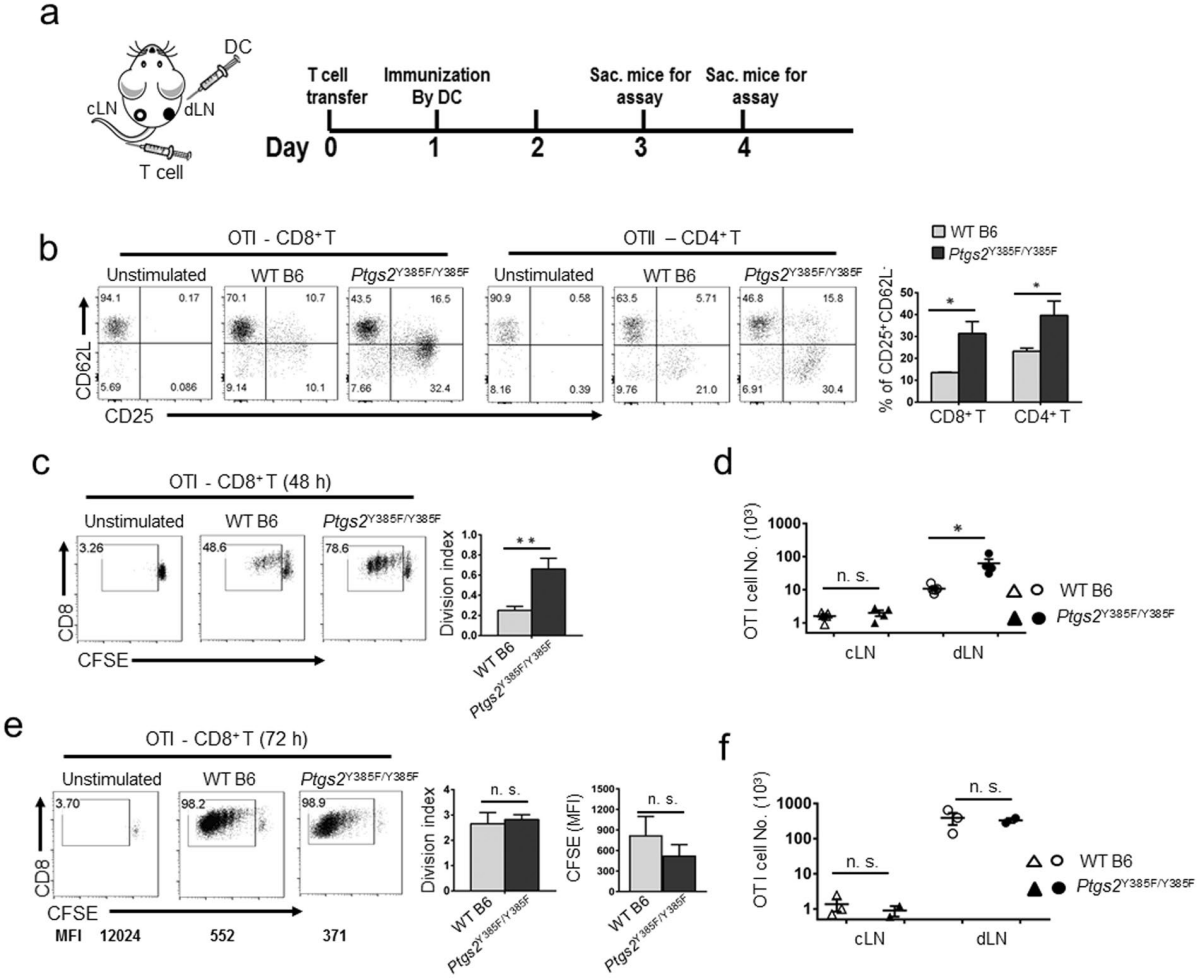
*In vivo* inactivation of *Cox-2* alleviates FRC-mediated suppression in the lymphoid tissues allowing more rapid T cell activation upon DC vaccine. (**a**) Schematic of adoptive transfer of OT-I/II T cells and DCs vaccine for *in vivo* OVA-antigen-specific T cell activation. CFSE labeled OT-I and OT-II T cells were transferred into WT C57BL/6 or *Ptgs2* ^Y385F/Y385F^ mice by i.v. injection. One day later, OT-I/II peptides loaded BMDCs were injected into the right rear paw of each mouse. (**b**) Mice were terminated 36 hrs post-DC vaccine and their LNs draining from the site of DC injection (dLN) and those opposite to the side of injection (cLN) were harvested for flow cytometry analysis. The upregulation of T cell activation markers, CD25 and CD62L, in OVA-specific OT-I cells in WT C57BL/6 and *Ptgs2* ^Y385F/Y385F^ mice were examined. (**c**) and (**e**) T cell proliferation indicated as CFSE dilution of OT-I T cells at 48 hrs and 72 hrs post-vaccine was determined via FACS. Division index and CFSE MFI of total OT-I cells was calculated and presented. (**d**) and (**f**) Total OT-I number in dLN and cLN of WT C57BL/6 and *Ptgs2* ^Y385F/Y385F^ mice were determined. Data (**b**–**f**) are representative of three independent experiments, with a pool of 3–5 mice for each different time points (mean ± SEM; *P < 0.05, **P < 0.01, n.s. = not significant; Nonparametric Mann-Whitney test).

To evaluate the effects of COX-2/PGE_2_ pathway in FRCs of WT and *Ptgs2* ^*M*^ mice on modulating T cell responses to vaccination *in vivo*, we adoptively transferred OVA antigen specific CD8 OT-I or CD4 OT-II T cells to *Ptgs2* ^*M*^ or WT mice (ACT), followed with activation by injecting OT-I or OT-II peptide loaded or non-antigen loaded DCs as unstimulated control one day after ACT (Fig. 6a). Strikingly, 36 hours following DC vaccine, a significantly higher percentage of OT-I and OT-II T cells with activated phenotype of CD25 upregulation and CD62L downregulation was observed in *Ptgs2* ^*M*^ mice than those in WT mice (Fig. 6b). Similarly, OT-I and OT-II T cells in the LNs draining from the vaccine site of *Ptgs2* ^*M*^ mice proliferated and expanded to about 5 to 10-fold higher in numbers than those in WT mice at 48 hours post-vaccine (Figs 6c,d and S6a,b). In contrast, no differences in the numbers of OT-I or OT-II cells within the non-draining LNs were observed between WT and *Ptgs2* ^*M*^ mice (Fig. 6d and S6b), which verified the antigen-specific activation. Interestingly, by 72 hours post-activation, the differences of OT-I or OT-II T cell proliferation and expansion between *Ptgs2* ^*M*^ and WT hosts were reduced (Figs 6e–f and S6c,d). Together, these results demonstrated that inactivation of the COX-2/PGE_2_ axis in FRCs engenders a less immunosuppressive environment that allows easier initiation and proliferation of T cells. Importantly, these results also confirm that different from T cell dysfunction, FRC-mediated immune suppression via COX-2/PGE_2_ axis can be overcome by strong activation stimuli, which is essential for host defense against environmental threats. Therefore, during homeostasis, hyperactive COX-2/PGE_2_ axis in FRCs serves to restrain T cell responsiveness to minimize unnecessary T cell activation and harmful onset of autoimmunity without compromising host defense against exogenous invaders.

### Hyperactivated COX-2/PGE_2_ axis is also an intrinsic property of FRCs in human lymphoid tissues

**Figure 7:**
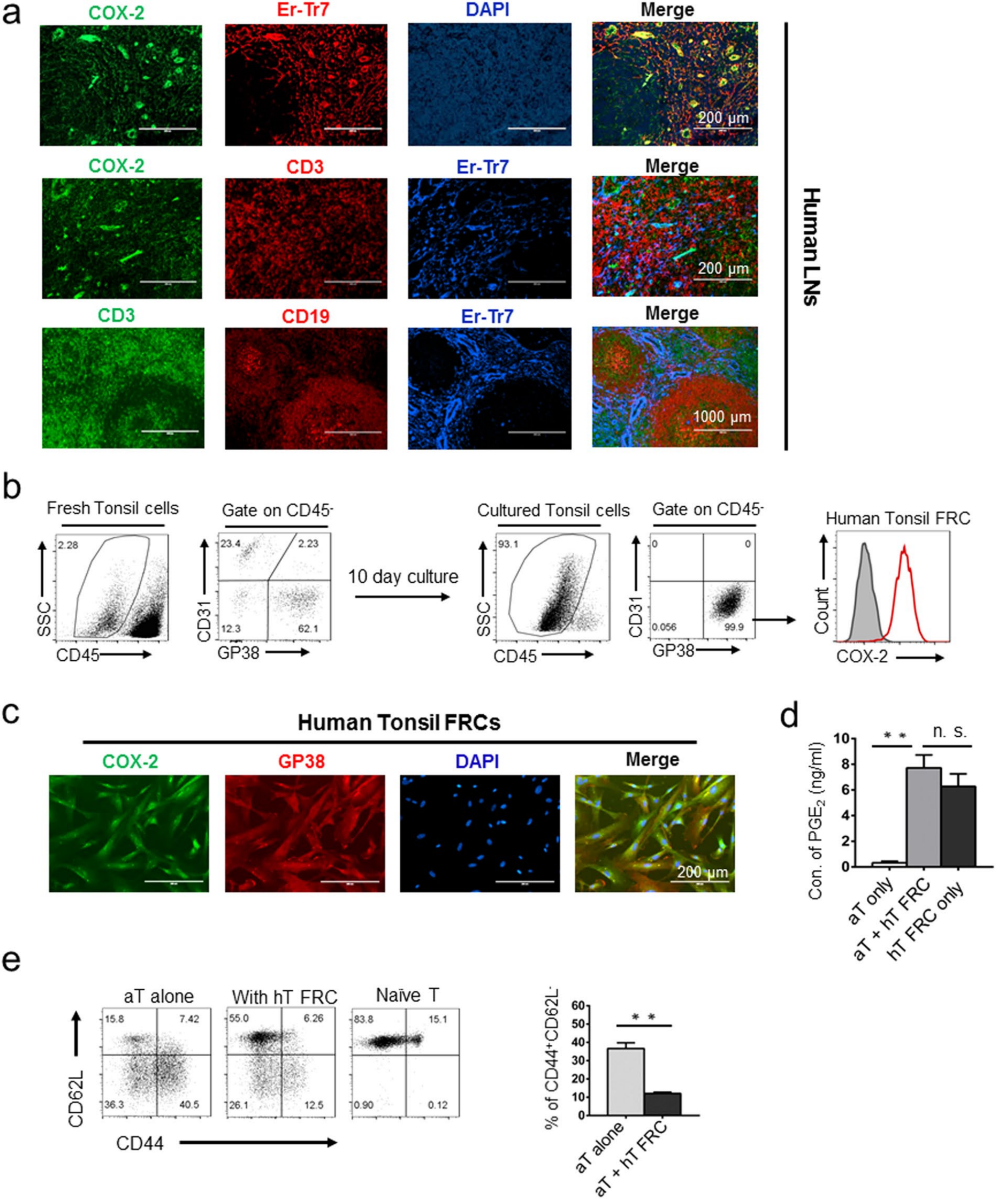
COX-2/PGE_2_ hyperactivity is implicated in fresh-FRCs of human lymphoid tissues. (**a**) Microscopy examination of COX-2 expression and distribution in a normal human lymph node. Scale bars, 200 μm (top two rows) and 1000 μm (bottom row), respectively. (**b**) FRCs in freshly isolated and cultured human tonsils as CD45^−^GP38^+^CD31^−^ cells were confirmed via flow cytometry analysis. COX-2 expression in FRCs was confirmed via FACS analysis following intracellular staining (gray histogram, isotype control). (**c**) Immunofluorescence staining of COX-2 (green), GP38 (red) and DAPI (blue) for cultured human FRCs from human tonsils. Scale bars, 200 μm. (**d**) PGE_2_ produced by the T cells or human tonsil FRCs was analyzed via ELISA. (**e**) Naïve CD8 T cells from WT mice were stimulated by anti-CD3/28 beads in the absence or presence of human FRCs from tonsils. T cell activation status was determined by the expression of CD62L and CD44 via FACS analysis. **P < 0.01, nonparametric Mann-Whitney test. Data are representative of two to three independent experiments with three replicates each. Error bars depict mean ± SEM.

To assess the clinical implication of our observed hyperactive COX-2/PGE_2_ pathway in murine SLN-FRCs, we examined COX-2 expression in stromal populations of human lymphoid tissues. Histological examination of human LNs via IF staining showed a high expression level of COX-2 in the ER-TR7^+^ stromal population (Fig. 7a, top panels), which appeared to be densely distributed in regions with abundant CD3^+^ T cells (Fig. 7a, middle panels). Further examination of CD3 and CD19 cell distribution that outlined T cell zones and germinal centers (Fig. 7a, bottom panels), respectively, supported the notion that the high COX-2 expressing cells were mostly located in the stromal network of interfollicular T cell area, reminiscent of the FRC-network in the T cell zone of mouse LNs (Figs 4b and 7a).

To demonstrate the activity and function of COX-2/PGE_2_ axis in FRCs of human lymphoid tissues, we purify FRCs from human tonsils following a previously established protocol^49^ with modest modifications as the aforementioned procedures used in mouse SLN-FRCs. As expected, human tonsils consisted of phenotypically comparable FRCs, identified as CD45^−^GP38^+^CD31^−^ cells, as those in mouse SLN-FRCs (Fig. 7b). These human FRCs expressed high levels of COX-2 (Fig. 7b and c) and produced abundant amount of PGE_2_ either in the absence or presence of T cells (Fig. 7d). Functional analysis confirmed that they markedly suppressed the activation of murine T cells stimulated by anti-CD3/CD28 beads (Fig. 7e). Altogether, our results demonstrate the parallel phenotype and function of hyperactive COX-2/PGE_2_ pathway in both human and mouse FRCs in the lymphoid organs during homeostasis for maintaining T cell peripheral tolerance in a non-antigen specific manner.

## Discussion

It has been increasingly appreciated that FRCs in secondary lymphoid organs play important roles in supporting immune cell survival, maintaining peripheral tolerance, and regulating host immune responses via multiple mechanisms^10, 11, 13, 36, 50, 51^. This study demonstrates that hyperactive COX-2/PGE_2_ pathway in FRCs is another previously unrecognized mechanism of maintaining peripheral T cell tolerance during homeostasis. Under physiological conditions, this high level of COX-2 establishes a suppressive milieu in the T cell zone of lymphoid tissues by PGE_2_ production. This leads to elevated T cell activation threshold and reduces T cell sensitivity to environmental cues, thereby providing another means of restraining unnecessary T cell activation and preventing harmful onset of autoimmunity. Also agreeing with our observations, microarray database analyses from a previously published report^52^ revealed that *Cox-2* (*Ptgs2*) expression is among one of the highly expressed genes in fresh SLN-FRCs. Interestingly, an early report also implied a potential involvement of COX-2/PGE2 pathway in FRC-mediated T cell suppression although the documented inhibition was modest, likely due to the differences in culture conditions and assay systems^15^. Importantly, our study confirms the COX-2/PGE_2_ hyperactivity in FRCs of human lymphoid tissues, suggesting the existence of a common mechanism shared between mice and humans for maintaining T cell tolerance during homeostasis by PGE_2_.

Fundamentally, this FRC-mediated T cell suppression via the COX-2/PGE_2_ pathway during homeostasis clearly differs from the previously identified mechanisms of FRC-induced tolerance, including PTA presentation-mediated deletion of self-reactive T cells and iNOS-NO-mediated inhibition of T cell proliferation during inflammation and/or towards late phase of T cell activation, in the following aspects: (1) hyperactive COX-2/PGE_2_-induced FRC-mediated immune suppression serves as a general mechanism to prevent accidental activation of self-destructive T cells during homeostasis in a non-antigen specific manner; (2) this COX-2/PGE_2_ induced T cell suppression in the lymphoid tissues is reversible, as demonstrated in our observed changes in T cell sensitivity to activation stimuli and activation kinetics following changes in the FRC-environment where T cells reside (Figs 5 and 6); (3) this FRC-mediated suppression via COX-2/PGE_2_ axis imposes a modest restraint of T cell activation that is more immunologically relevant and evident during homeostasis and the early phase of T cell activation. In contrast, the NO-iNOS pathway in FRCs is only active after T cells are fully activated to further restrict T cells from over-activation, thereby facilitating the termination of activation and re-establishment of homeostasis^13, 52, 53^; and (4) importantly, this level of FRC-mediated suppression via the COX-2/PGE_2_ axis can be overcome by strong activation stimuli associated with pathogenic threats. Once the strong and persistent activation stimuli overcome this COX-2/PGE_2_-mediated suppression, the early effects of PGE2-associated suppression on T cell activation are alleviated, which explains why the differences in T cell activation and proliferation in WT and *Ptgs2* ^*M*^ mice were diminished by 72 hours post-activation (Figs 6e,f and S6c,d). Together, these findings demonstrate a clear distinction between FRC-associated PGE_2_-mediated elevation of T cell activation threshold and T cell dysfunction under pathological conditions.

It is generally believed that COX-2 is induced by inflammatory stimuli/cytokines to subsequently limit further immune activation and minimize potential collateral damage, whereas COX-1 is constitutively expressed and is responsible for basal low level of PGE_2_^31, 32, 54–56^. Mechanistically, it is not yet entirely clear how the basal COX-2/PGE_2_ activity in FRCs is elevated and maintained at the extremely high levels. We postulated that cytokines, growth factors, or other environmental cues in the LNs actively participate in regulating the COX-2/PGE_2_ activity because TNF-α, IL-6 and LPS have all been shown to upregulate *Cox-2* expression^47, 54, 56–58^. It is very likely that more than one factors are involved in this observed COX-2/PGE_2_ hyperactivity in FRCs, which will be the subject of future investigations. Interestingly, our comparative analysis of levels of COX-2/PGE_2_ in FRCs, tumors, and MDSCs suggests that on per cell basis, the FRC-COX-2/PGE_2_ activity is at least as high as, if not higher than, that of tumors and tumor-associated MDSCs. Due to the significant differences in their relative abundancy, i.e. FRCs being less than 0.1% of the LN cellular constituents as compared with the dominant presence of tumors and high abundancy of MDSCs within the TME, it is not surprising that under physiological conditions, FRCs only impose modest T cell suppression by elevating T cell activation threshold as compared with the “global” COX-2/PGE_2_ activity in the TME imposed by tumors and MDSCs under pathological conditions, which compels detrimental immunosuppression on tumor infiltrating T cells^23, 33^. It is also yet to be determined whether tumors and MDSCs share a similar mechanism with FRCs in regulating and maintaining high activity of COX-2/PGE_2_ pathway.

In summary, our study for the first time illustrates another previously unidentified mechanism of FRCs in both mice and humans that maintains peripheral T cell tolerance by fine-tuning T cell activation threshold during homeostasis. Nevertheless, this FRC-mediated suppression is different from T cell dysfunction in that it can be overcome by a stringent activation signal so that proper T cell responsiveness against environmental threats is ensured. Therefore, during homeostasis the FRC-COX-2/PGE_2_ serves as a “gate-keeper” in restraining T cells from unnecessary activation in a non-antigen specific manner to prevent accidental activation of self-damaging T cells.

## Materials and Methods

### Mice and human specimens

C57BL/6 mice aged 4–6 weeks were purchased from Charles River (Wilmington, MA). *Ptgs2* ^Y385F/Y385F^ mice, OVA-specific CD8 (OT-I), and CD4 (OT-II) TCR transgenic mice were obtained from the Jackson Laboratory (Bar Harbor, ME). All mice were maintained under specific pathogen free conditions in the Animal facility of the Augusta University following protocols approved by the Augusta University Institutional Animal Care and Use Committee. De-identified frozen human lymph nodes and fresh tonsils were obtained from Georgia Cancer Center Biorepository Bank and Ochsner Clinic Foundation, respectively, following protocols approved by Institutional Review Board committees at both institutions. Informed consent was obtained from all subjects providing fresh tissues. All experimental methods were carried out in accordance with relevant guidelines and regulations.

### Antibodies and reagents

The following antibodies were used for flow cytometry analysis: anti-CD3 (UCHT1), anti-CD4 (RM4-5), anti-CD8α (53–6.7), anti-CD11b (M1/70), anti-CD19 (HIB19), anti-CD25 (PC61), anti-CD31 (MEC13.3), anti-CD44 (IM7), anti-CD45.1 (A20), anti-CD45.2 (104), anti-CD69 (H1.2F3), anti-CD90.1 (OX-7), anti-CD90.2 (53-2.1), anti-VCAM-1 (429), and biotin-anti-mouse CD3 (145-2C11), all from BD Biosciences (San Jose, CA); anti-CD54 (YN1/1.7.4), anti-CD62L (MEL-14), anti-IFN-γ (XMG1.2), anti-IL2 (ALF-161), anti-Sca-1 (D7), anti-Ter-119 (Ter-119) and anti-pZAP-70/SYK (n3kobu5) all from eBioscience (San Diego, CA); anti-CD140a (APA5), anti-CD140b (APB5), ant-human (NC-08) and anti-mouse gp38 (8.1.1) all from Biolegend (San Diego, CA). Anti-COX-2 (D5H5) was from Cell signaling (Boston, MA); Alexa Fluor 488–conjugated goat anti-rabbit (A11008) was from Life Technologies (Carlsbad, CA). Functional-grade purified anti-CD3ε (17A2) was from Biolegend. The following chemical reagents and inhibitors were used: 1400 W and L-NMMA (NG-monomethyl-l-arginine); both from Calbiochem (Billerica, MA). Mouse T - activator CD3/CD28 Dynabeads, streptavidin, Indo-1 AM and CFSE [5(6)-carboxyfluorescein diacetate, succinimidyl ester] were all from Life Technologies. PGE_2_ (Prostaglandin E2), SC-236 and Indomethacin were all from Cayman Chemicals (Ann Arbor, MI). APCP (Adenosine 5′-(α,β-methylene)diphosphate), ZM241385 and PSB1115 were all from Sigma (St. Louis, MO). LSRII flow system (BD Biosciences) was used for flow cytometry and data processed with FlowJo (Tree Star Inc., Ashland, OR).

### SLN stromal cell isolation, *ex vivo* expansion, and FRC purification

Murine draining axillary, brachial and inguinal SLNs pooled from multiple mice were dissected, minced into small pieces in DMEM supplemented with 10% fetal bovine serum (FBS) and 1% penicillin-streptomycin (Life Technologies). They were digested with 0.8 mg/ml Dispase, 0.2 mg/ml Collagenase P, and 0.1 mg/ml DNase I (all from Roche, Basel, Switzerland) at 37 °C with shaking for 60 min. At 15 min intervals, digested tissues were pipetted to facility dissociation and dispersed single cells were collected and kept on ice similar to previously described^45^. At the end of 60 min digestion, all dispersed cells were pooled. For fresh FRC sorting, the digested SLN cell suspension was filtered through a 70 μm strainer and stained with biotinylated anti-mouse CD4 (RM4-5), CD8α (53-6.7), CD11b (M1/70), CD19 (1D3) and Ter-119 (Ter-119) antibodies, followed with EasySep^TM^ Mouse Streptavidin RapidShere and EasySep^TM^ (STEMCELL Tech., Vancouver, Canada) Magnet-based negative selection. These negatively selected cells were stained with CD45.2 (104), CD31 (MEC13.3), and gp38 (8.1.1) for FACsorting CD45^−^ CD31^−^ gp38^+^ FRCs via FACSAria (BD Biosciences) using low pressure and 100 μm nozzle. For total stromal cell culture and expansion, following SLN digestion, cell suspension was directly plated in DMEM medium and nonadherent cells removed 48 h later. After 5–10 day culture, adherent cells, usually consisting of 5–10% CD11b^+^ myeloid cells and ~90% CD11b^−^CD45^−^ stromal cells, were harvested and purified by FACsorting as CD45^−^CD11b^−^CD31^−^gp38^+^ FRCs with a purity of >95%.

### Histology and fluorescent immunohistochemistry

Lymph nodes were snap frozen in liquid nitrogen. Cryosections were fixed in ice-cold acetone and pre-incubated in blocking solution (1% BSA and 5% goat serum in PBS) for 30 minutes at room temperature (RT). Primary antibody binding was performed overnight at 4 °C, followed with secondary antibody for 2 h at RT. To visualize COX-2 in cultured FRCs, cells were plated in 4-well slide chamber (Millipore) overnight, fixed in 4% Paraformaldehyde, and permeabilized by 0.1% Triton X-100 (SIGMA) for 5 min at RT. The subsequent staining was similar to the aforementioned procedures. Images were acquired using Evos (Life Technologies) fluorescent microscope and resized using Adobe Photoshop.

### Real-time reverse transcription–PCR

Total RNA was harvested using Qiagen RNeasy Kits. Reverse transcription was performed with 1 μg of total RNA using random hexamers as primers and Superscript II reverse transcriptase (Life Technologies). Real-time PCR were carried out following standard protocols with primers purchased from Integrated DNA Technologies (Coralville, IA) using a BioRad CFX384 (BioRad Life Science Research, Hercules, CA). Primers used for the amplification of murine *Cox-2* are as follows (forward primer: CTC CGT AGA AGA ACC TTT TCC A; reverse primer: CCT TCT CCA ACC TCT CCT ACT). *β-actin* primers, forward primer: GAC TCA TCG TAC TCC TGC TTG; reverse primer: GAT TAC TGC TCT GGC TCC TAG.

### T cell co-culture with FRCs

Purified FRCs were seeded in a 24 or 48-well plate at 4 × 10^4^/well or 2 × 10^4^/well, respectively. Following overnight adhesion, 1 × 10^6^ or 5 × 10^5^ naïve purified T cells (STEMCELL EasySep^TM^ negative selection), either CFSE-labeled or unlabeled, in 0.5–1 ml RPMI-1640 medium (10% FBS) in the presence or absence of CD3/CD28 Dynabeads (Life Technologies) were added to each well. Early T cell activation was analyzed 6–15 h post-stimulation and T cell proliferation as CFSE dilution was examined 2–3 days post-stimulation. For reconditioning naïve T cells in culture in the absence or presence of FRC, 30 ng/ml recombinant murine IL-7 was added to the culture medium to maintain viability. For the transwell culture, T cells and activation beads were added to the upper chamber of a transwell (pore size of 0.4 μm, Corning^TM^, Corning, N.Y.). For antigen-specific T cell activation, OT-I loaded BM-derived DCs (1 × 10^5^) were added to 1 × 10^6^ Ova-specific OT-I T cells, with or without 4 × 10^4^ FRCs/well in 24 well plate.

### BM-MDSC and BM-DC differentiation from BM culture

Bone marrow cells were isolated from femurs and tibias of mice. For MDSCs differentiation, BM cells were resuspended at 2 × 10^5^/ml in DMEM medium with 10% FBS and cultured in the presence of 100 U/ml GM-CSF and 40 ng/ml IL-6 for 4 days. Suspension cells were collected and used for RNA harvesting. BMDCs were prepared from a 8-day culture of bone marrow cells in RPMI medium containing 5% FBS and 1000 U/ml recombinant murine GM-CSF at the concentration of 1 × 10^6^ cells/ml. Fresh medium containing GM-CSF was replaced every 2 days. On day 6, loosely adhered cells were collected and re-plated at 5 × 10^5^/ml in 100 mm dish in the presence of GM-CSF. Mature BM-DC were harvested on day 8 for subsequent functional analyses and RNA harvesting.

### Human tonsil stromal cell isolation, expansion and cell sorting

Human tonsils were similarly processed as the aforementioned procedures for mice SLN stromal cell culture. After 7–10 d, adherent cells, mainly contained 10% CD45^+^ cells and 90% FRCs, were harvested and purified by FACsorting as CD45^−^CD31^−^gp38^+^ FRCs with a purity of >95%.

### Calcium mobilization

Naïve T cells were incubated for 30 min at 37 °C with 5 μM Indo-1 AM. Following removal of Indo-1 by washing with RPMI-1640 medium, T cells were labeled with biotinylated anti-CD3 for 15 min. The T cell baseline fluorescence (Indo-violet & Indo-blue) was first recorded for 1 min, followed with rapid CD3-ligation by adding 10 μg/ml streptavidin and simultaneous recording of fluorescence intensity (Indo-violet & Indo-blue) for additional 2–3 min. Positive control stimulation was performed using 1 μM ionomycin.

### pZAP70 by flow cytometry

Fresh or cultured naïve T cells were incubated with soluble anti-CD3ε (17A2) to stimulate for 30 min and then immediately fixed with 2% formaldehyde for 15 min at 37 °C. After washing with permeabilization buffer (eBioscience), the cells were stained with anti-pZAP70 (1:50 dilution) in 100 μl permeabilization buffer at 4 °C for 30 min, washed and analyzed by flow cytometry.

### Western blotting

Cell lysates containing 100 μg protein/sample were separated with NuPAGE 10% Bis-Tris gels (Invitrogen) and transferred onto PVDF membranes (Life Technologies). The membranes were blotted with antibodies against COX-2 (Cell signaling), followed by a secondary antibody conjugated with horseradish peroxidase (Santa Cruz Biotechnology, Santa Cruz, CA). The blots were visualized using Super Signal® West Dura Chemiluminescent substrates (Pierce Biotechnology, Rockford, IL) according to the manufacturer’s instructions. Western blot digital images were obtained using Fujifilm LAS-300 Imager.

### T cell activation *in vivo*

CFSE-labeled naive OT-I CD45.1^+^ T or OT-II CD90.1^+^ T cells were intravenously transferred into C57BL/6 or *Ptgs2* ^Y385F/Y385F^ mice at 1 × 10^6^/mouse. Twenty-four hour post-T cell transfer, each mouse received 5 × 10^5^ BM-DCs with or without OT-I or OT-II peptide loading in the right hind paw. Thirty-six to seventy-two hours post-DC-vaccine, draining LNs (popliteal and inguinal) and non-draining LNs (left flank of SLNs) were collected, single-cell suspensions were prepared and stained for flow cytometry.

### PGE_2_ quantification in culture medium

Prostaglandin E2 express EIA kit (Cayman; 500141) was use to determinate the concentration of PGE_2_ in culture medium. The procedures of the ELISA exactly follow the manual description.

### Measurement of nitric oxide

Nitric oxide concentrations in culture supernatants were determined using the Griess Reagent System (Promega, Madison, WI). Briefly, samples (50 μl) were added to the 96 well microtiter plates and mixed with 50 μl of 1% sulfanilamide and 50 μl of 0.1% naphthylethylenediamine in 2.5% H3PO4 at RT for 5–10 min. The absorbance at 530 nm was measured and compared with that of a serial dilution of standard control of NaNO_3_.

### Statistical analysis

Statistical analysis was assessed by two-tailed unpaired nonparametric Mann-Whitney test using Prism 6.0 software (Graph-Pad). A *p* value of < 0.05 was considered significant.

## Electronic supplementary material

Supplemental figures
