# Supplementary material for: Fibroblastic reticular cells of the lymphoid tissues modulate T cell activation threshold during homeostasis via hyperactive cyclooxygenase-2/prostaglandin E2 axis
Source: Sci Rep. 2017 Jun 13;7:3350. doi: 10.1038/s41598-017-03459-5 (PMC5469856; doi:10.1038/s41598-017-03459-5)
Supplement: Supplementary file 1 — Supplemental figures [file 41598_2017_3459_MOESM1_ESM.pdf]

Fibroblastic reticular cells of the lymphoid tissues modulate T cell activation threshold during homeostasis via hyperactive cyclooxygenase-2/prostaglandin E_2 axis

Miao Yu[1†], Gang Guo[1†], Xin Zhang[2], Li Li[2], Wei Yang[1, 3], Roni Bollag[4], Yan Cui[1*]

[1]Department of Biochemistry and Molecular Biology, Cancer Immunology, Inflammation & Tolerance Program, Georgia Cancer Center, Augusta University, Augusta, GA 30912, USA

[2]Institution of Translational Research, Gayle & Tom Benson Cancer Center, 1N505A, Ochsner Clinic Foundation, 1514 Jefferson Highway, New Orleans, LA 70121

[3]Current address: Department of Immunology, College of Basic Medical Sciences，Norman Bethune Health Science Center, Jilin University. 126 Xinmin Avenue, Changchun 130021, China

[4]Tumor Tissue and Serum Biorepository, Georgia Cancer Center, Augusta University, Augusta, GA 30912, USA

Supplementary information:

Supplementary Figures 1- 6

Original Western blot images of Fig. 5g (Supplementary Figure 7).

Original Western blot images of Fig. 4Sd (Supplementary Figure 8).

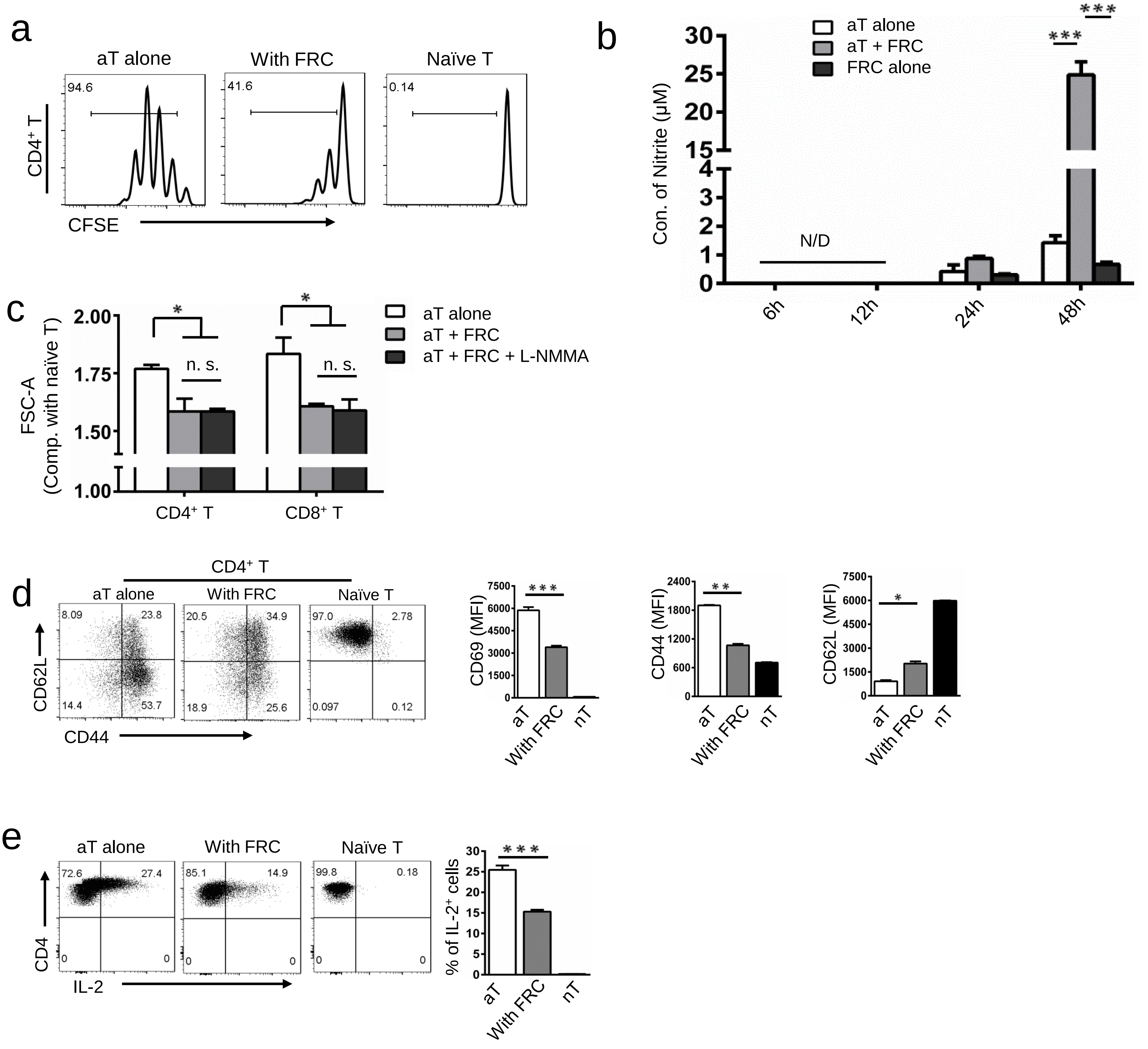

Supplementary Figure 1. SLN-FRCs inhibit T cell proliferation. (a) CFSE - labeled T cells were stimulated by anti-CD3/CD28 beads in the absence or presence of SLN-FRCs. T cell proliferation was assessed by flow cytometry as CFSE dilution 72 hrs after activation. The percent of divided cells was indicated in each plot. (b) Nitric oxide concentration in the supernatant of T cell and FRC culture following T cell activation from 6 to 48 hrs was determined via ELISA. N/D, not detectable. (c) Flow cytometry-based analysis of changes in size (FSC-A) of $CD4^+$ and $CD8^+$ T cells 20 hrs post-activation in the absence or presence of FRCs, without or with the NOS inhibitor L-NMMA (400 μM (c) Naïve CD4 T cells were stimulated by anti-CD3/CD28 beads for 15 hrs, in the absence or presence of SLN-FRCs. T cell activation was assessed by flow cytometry as changes in surface expression of CD69, CD62L, and CD44. Numbers in quadrants indicate cell percentages. MFI values of CD69, CD44 and CD62L are summarized in bar chart. (d) Following T cell activation by to anti-CD3/CD28 beads in the absence or presence of FRCs for 16 hrs, brefeldin A (BFA, 5 μg/ml) was added to CD4 T cells for 4 hrs. CD4 T cell production of IL-2 was determined via intracellular staining followed by FACS analysis. Data from three independent experiments are summarized in bar chart. ** $p < 0.05$, ** $p < 0.01$, *** $p < 0.001$ (nonparametric Mann-Whitney test). Data are representative of two to three independent experiments with three replicates each. Error bars depict mean ± SEM.

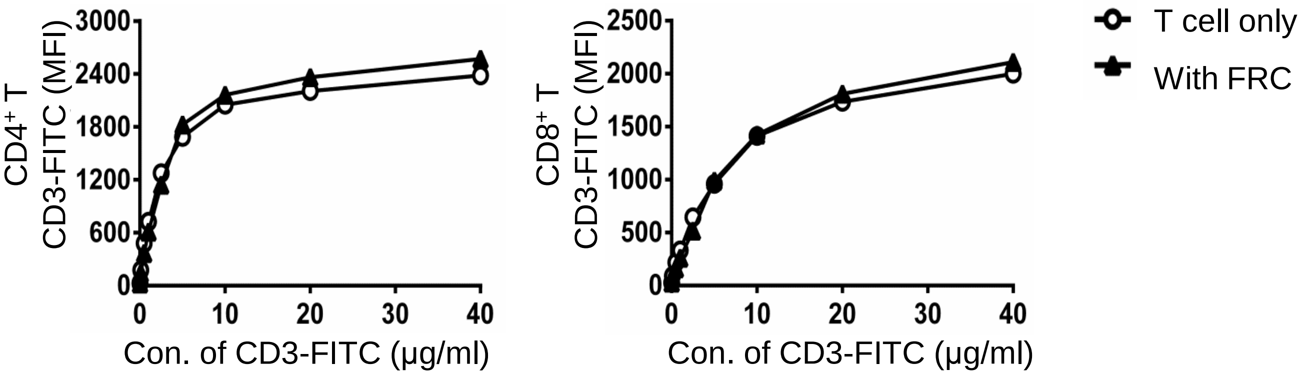

Supplementary Figure 2. The presence of SLN-FRCs do not interfer CD3-TCR binding to T cells. T cell accessibility to CD3 binding in those cultured in the absence or presence of SLN-FRCs was examined as the MFI of anti-CD3-FITC via flow cytometry. Data are representative of two to three independent experiments with three replicates each.

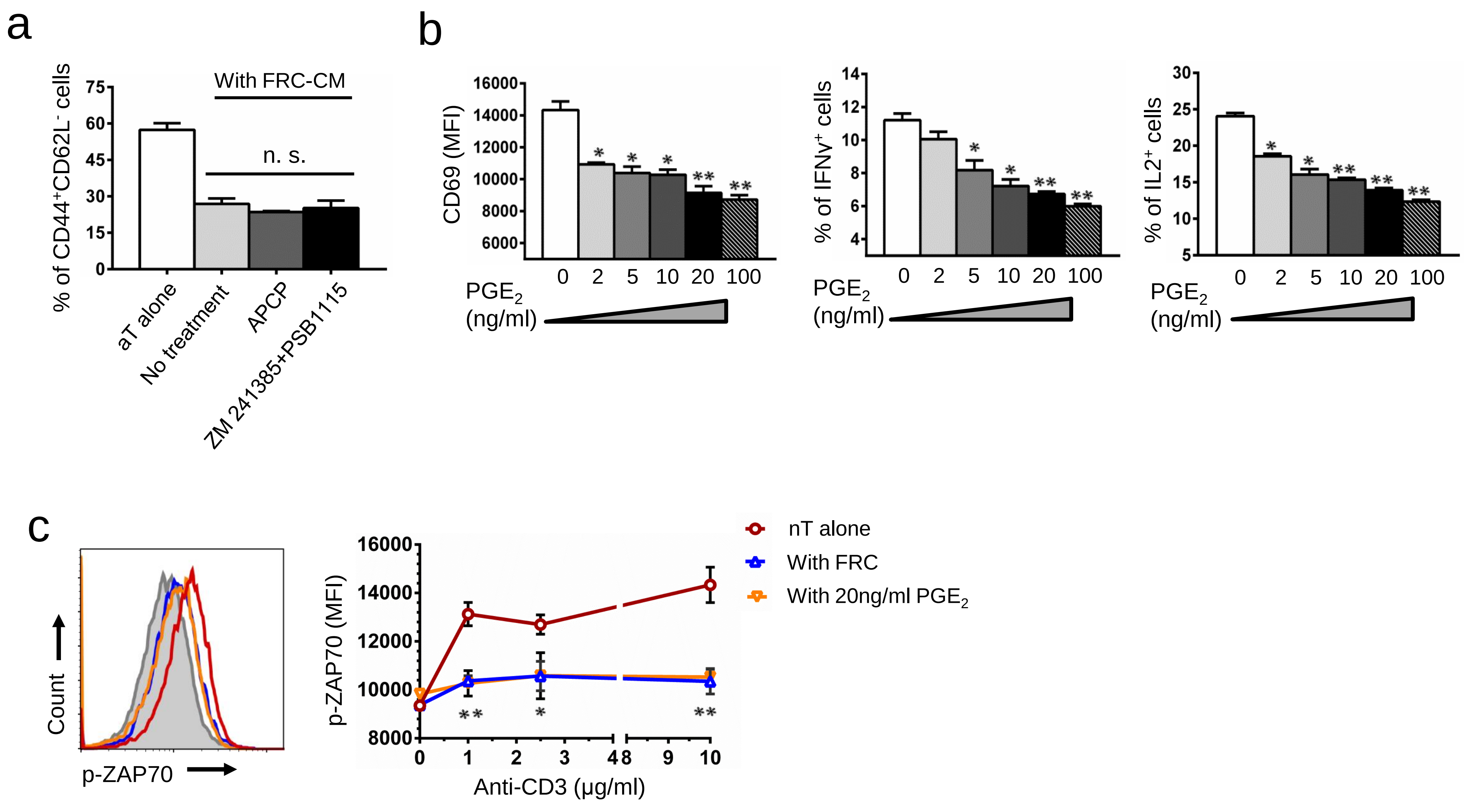

Supplementary Figure 3. FRCs inhibit T cell activation through heat-stable soluble factor PGE_2. (a) Various FRC-CMs were collected from cultured FRCs in the absence or presence of CD73 inhibitor APCP (10 μM) or A2A and A2B receptors antagonist of ZM241385 and PSB1115 (both at 1 μM). They were added to T cell activation at the dilution of 1:1; (b) T cells in the presence of various PGE_2 (0-100 ng/ml) were activated for 15 hrs. Their surface marker CD69 expression and effector cytokine IFN-γ and IL-2 production were assessed via flow cytometry. (c) The effects of 20 ng/ml PGE_2 (yellow) as compared with FRCs (blue) on TCR-ligation-induced ZAP70 phosphorylation were examined via flow cytometry. Representative histogram of ZAP70 phosphorylation upon 1 μg/ml anti-CD3 stimulation is shown (left), where unstimulated T cells (gray) were used as negative control. Data are representative of two to three independent experiments (mean ± SEM). ** $p < 0.05$, ** $p < 0.01$, compared with controls (nonparametric Mann-Whitney test).

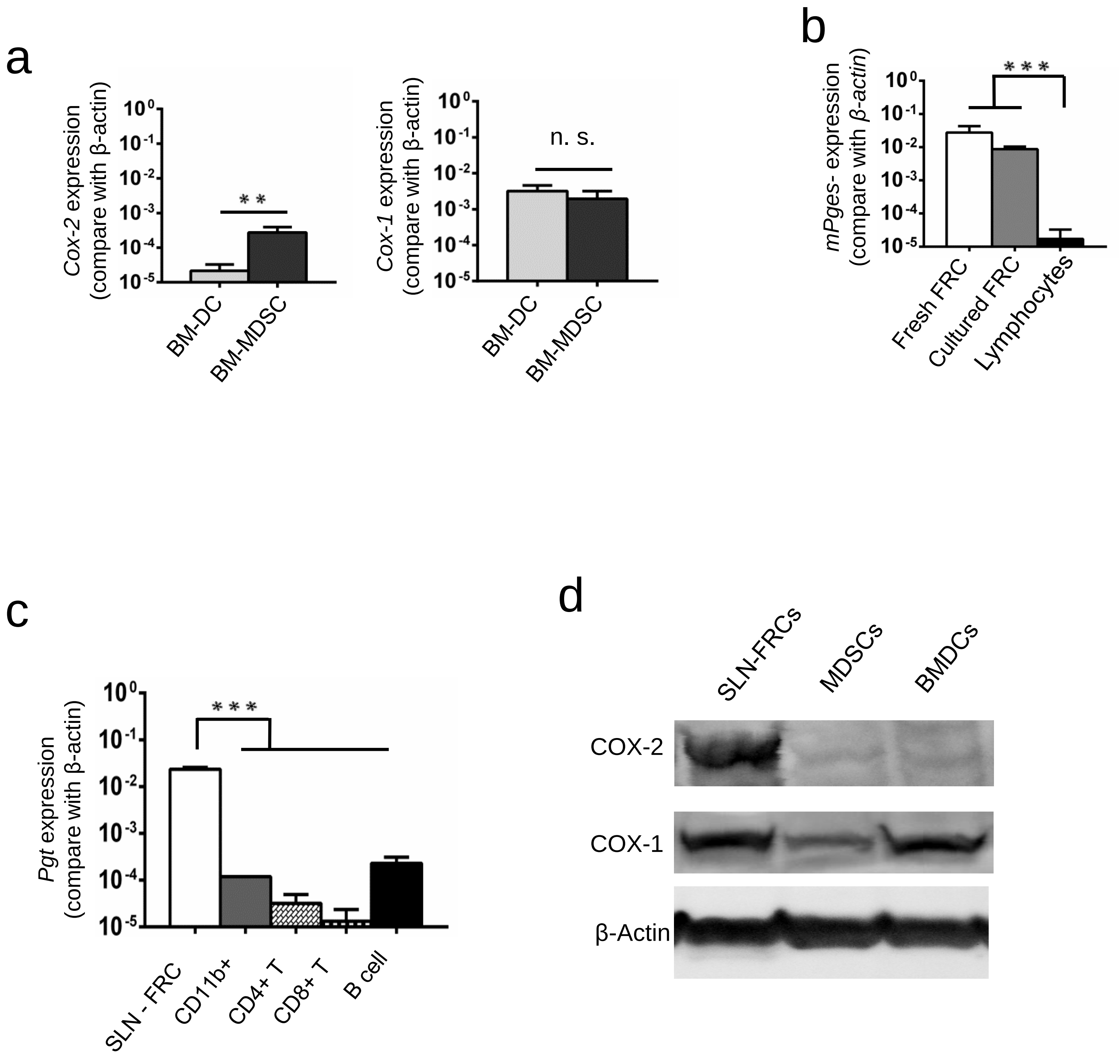

Supplementary Figure 4. COX-2/PGE2 pathway is highly activated in FRCs under homeostasis. (a) Comparative analysis of *Cox-2* and *Cox-1* mRNA expression in BM-DC and MDSC via quantitative real-time RT-PCR, which is normalized against the β-actin mRNA level (mean ± SEM). (b and c) Comparative analysis of *mPges-1* and *Pgt* mRNA expression in various SLN cells (mean ± SEM). (d) Cell lysates of cultured SLN-FRCs, BM-DC and MDSC were subjected to SDS-PAGE gel and Western Blotting analysis to determine COX-2 and COX-1 protein expression using b-actin as sample loading control. Cropped images of representative experimental results, where uncropped images are presented in Supplementary Figure 8. (a – c) Data are representative of two to three independent experiments (mean ± SEM). ** $p < 0.01$, *** $p < 0.001$, n.s. = not significant, nonparametric Mann-Whitney test.

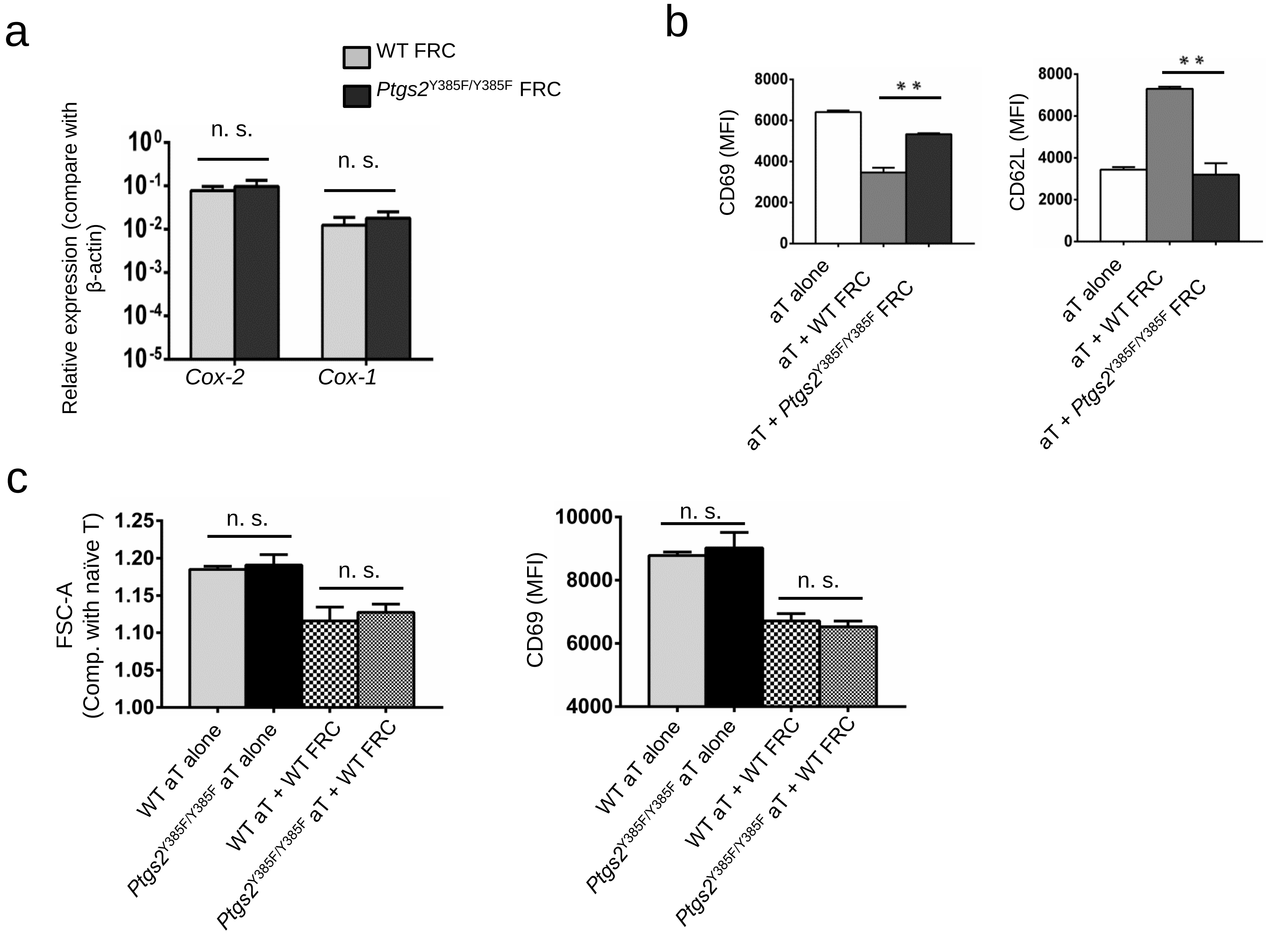

Supplementary Figure 5. Hyperactive COX-2/PGE_2 pathway in SLN-FRCs is associated with maintenance of T cell tolerance under homeostasis. (a) Comparative analysis of *Cox-2* and *Cox-1* mRNA expression via quantitative real-time RT-PCR. (b) Naïve T cells from WT C57BL/6 mice were activated in the absence or presence of SLN-FRCs from C57BL/6 WT or *Ptgs2*$^{Y385F/Y385F}$ mice. T cell activation was examined as MFI values of CD69 and CD62L expression. (c) T cells from WT C57BL/6 or *Ptgs2*$^{Y385F/Y385F}$ mice were cultured overnight in the absence or presence of FRCs from C57BL/6 mice. They were subsequently activated by anti-CD3/28 beads. T cell activation was determined via analysis of MFI values of FSC-A and CD69 expression. FSC-A, forward scatter – area. Data are representative of two to three independent experiments for CD4$^+$ T cells (mean ± SEM; ** $p < 0.01$, n.s. = not significant; nonparametric Mann-Whitney test).

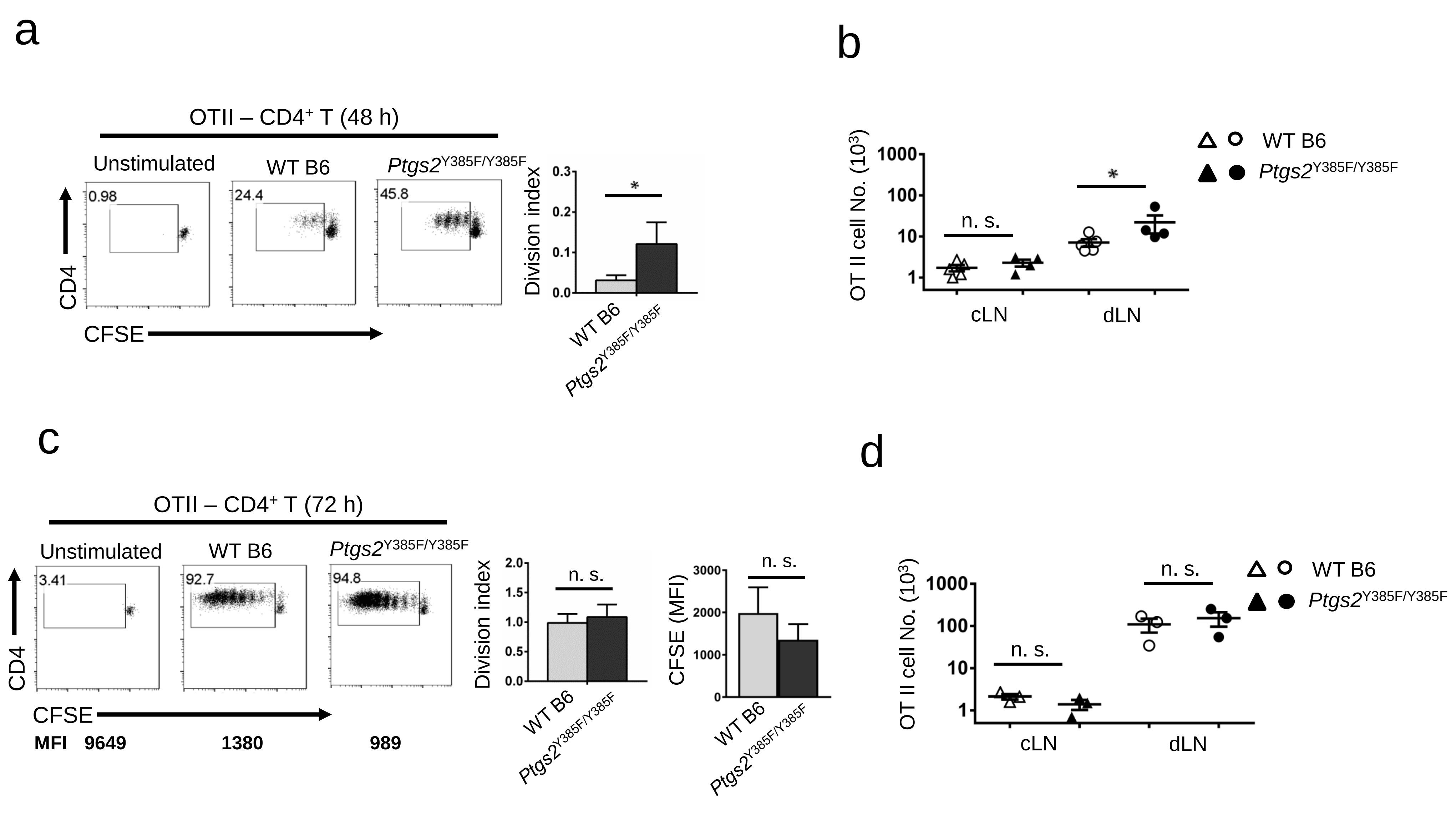

Supplementary Figure 6. *In vivo* inactivation of *Cox-2* alleviates FRC-mediated suppression in the lymphoid tissues allowing more rapid CD4 OT-II T cell activation upon DC vaccine. CFSE labeled OT-II T cells were transferred into WT C57BL/6 or *Ptgs2*$^{Y385F/Y385F}$ mice by i.v. injection. One day later, OT-II peptides loaded BMDCs were injected into the right rear paw of each mouse. Mice were terminated at 48 hrs (a and b) and 72 (c and d) hrs post-vaccine post-DC vaccine and their LNs draining from the site of DC injection (dLN) and those opposite to the side of injection (cLN) were harvested for flow cytometry analysis.(a) and (c) T cell proliferation indicated as CFSE dilution of OT-II T cells at 48 hrs and 72 hrs post-vaccine was determined via FACS. Division index and CFSE MFI of OT-II cells was calculated and presented. (b) and (d) Total OT-II number in dLN and cLN of WT C57BL/6 and *Ptgs2*$^{Y385F/Y385F}$ mice were determined. Data are representative of three independent experiments, with a pool of 3 - 5 mice for each different time points (mean ± SEM; * P < 0.05, n.s. = not significant; nonparametric Mann-Whitney test).

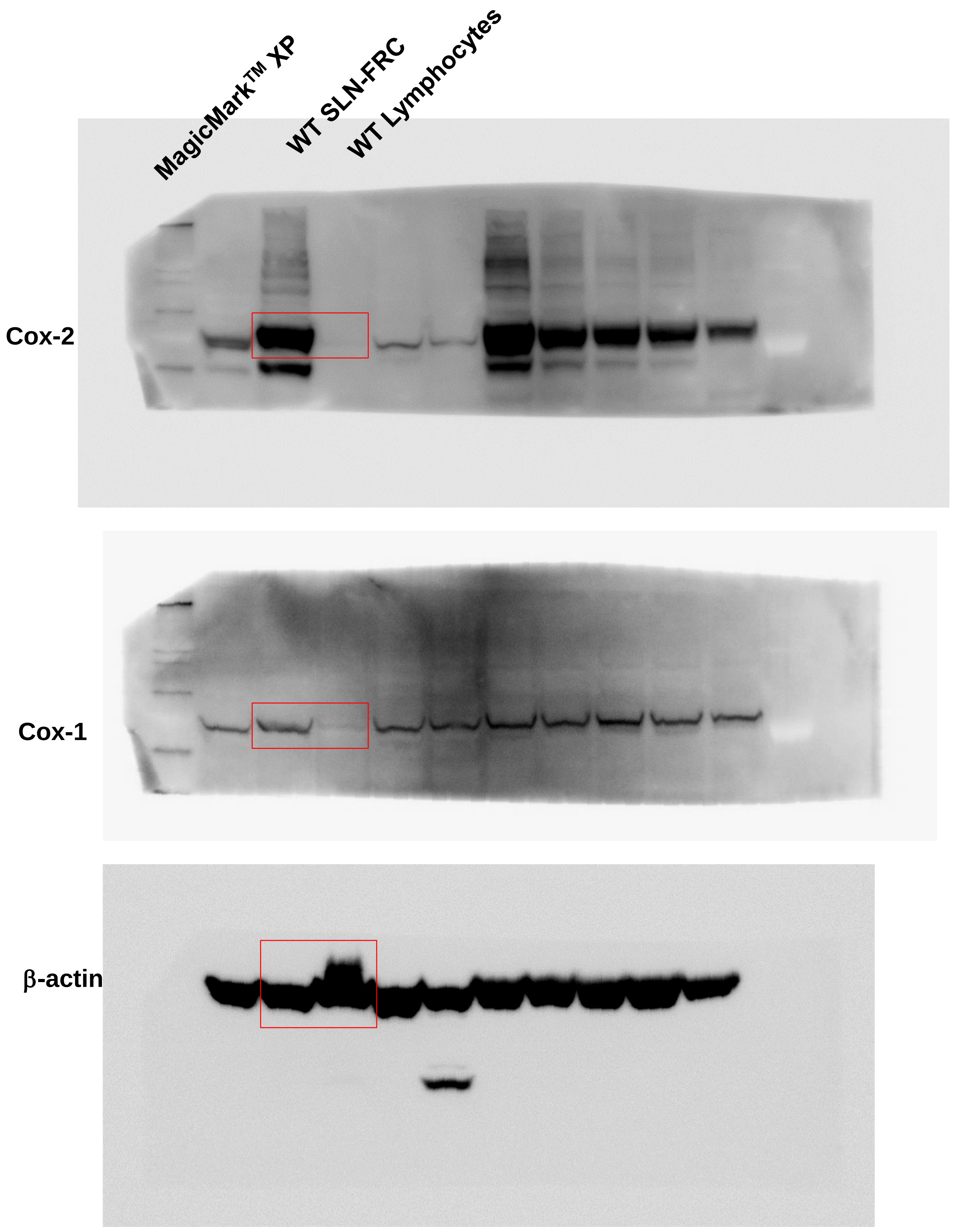

Figure 4g blots

Supplementary Figure 7

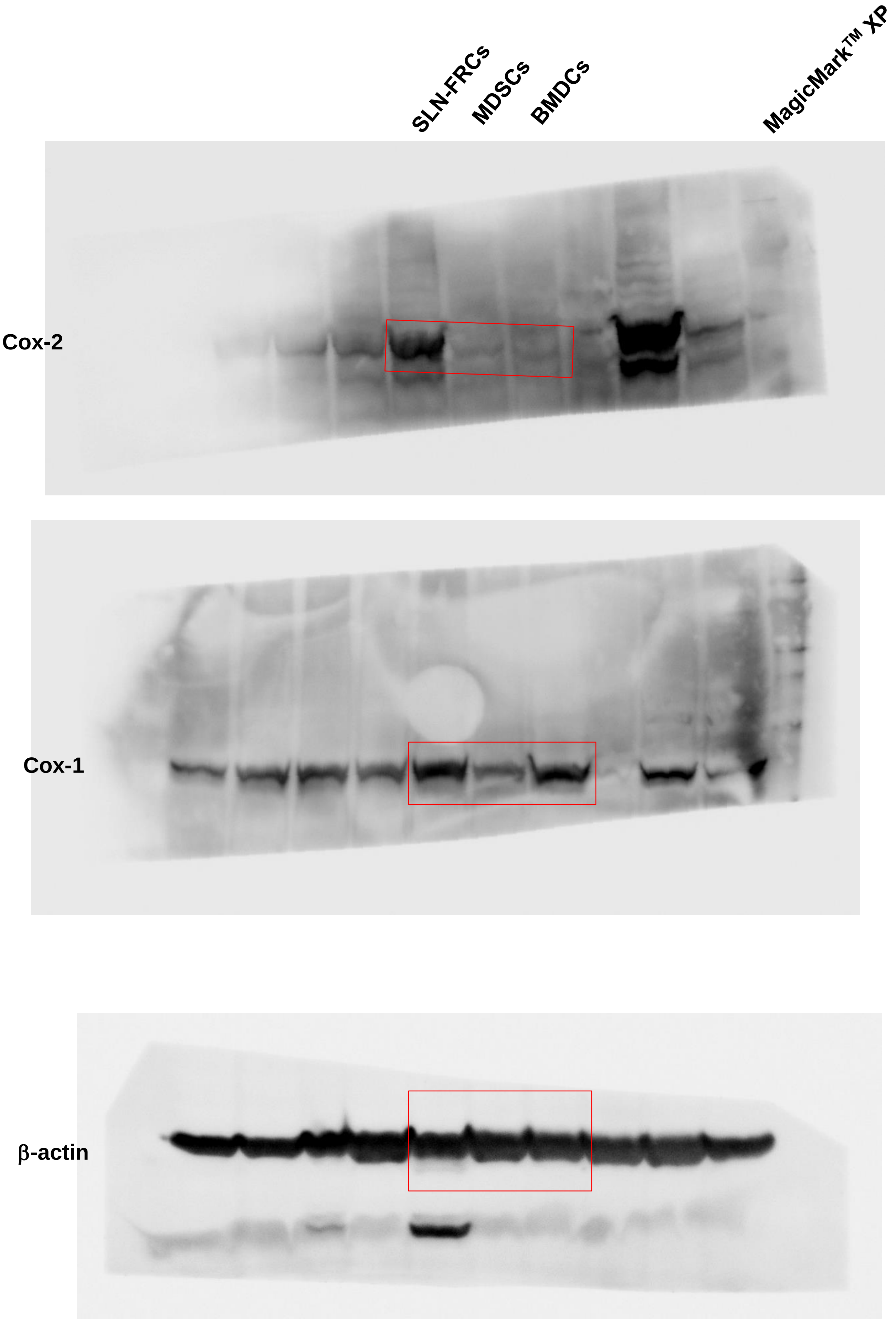

FigureS4d blots
